# Application of carbohydrate arrays coupled with mass spectrometry to detect activity of plant-polysaccharide degradative enzymes from the fungus *Aspergillus niger*

**DOI:** 10.1038/srep43117

**Published:** 2017-02-21

**Authors:** Jolanda M. van Munster, Baptiste Thomas, Michel Riese, Adrienne L. Davis, Christopher J. Gray, David B. Archer, Sabine L. Flitsch

**Affiliations:** 1 Fungal Biology and Genetics, School of Life Sciences, University of Nottingham, University Park, Nottingham NG7 2RD, UK; 2 Chemical Biology, Manchester Institute for Biotechnology, University of Manchester, 131 Princess Street, Manchester M1 7DN, United Kingdom; 3 School of Chemistry, University of Nottingham, University Park, Nottingham NG7 2RD, UK

## Abstract

Renewables-based biotechnology depends on enzymes to degrade plant lignocellulose to simple sugars that are converted to fuels or high-value products. Identification and characterization of such lignocellulose degradative enzymes could be fast-tracked by availability of an enzyme activity measurement method that is fast, label-free, uses minimal resources and allows direct identification of generated products. We developed such a method by applying carbohydrate arrays coupled with MALDI-ToF mass spectrometry to identify reaction products of carbohydrate active enzymes (CAZymes) of the filamentous fungus *Aspergillus niger*. We describe the production and characterization of plant polysaccharide-derived oligosaccharides and their attachment to hydrophobic self-assembling monolayers on a gold target. We verify effectiveness of this array for detecting exo- and endo-acting glycoside hydrolase activity using commercial enzymes, and demonstrate how this platform is suitable for detection of enzyme activity in relevant biological samples, the culture filtrate of *A. niger* grown on wheat straw. In conclusion, this versatile method is broadly applicable in screening and characterisation of activity of CAZymes, such as fungal enzymes for plant lignocellulose degradation with relevance to biotechnological applications as biofuel production, the food and animal feed industry.

The availability of well-characterised, affordable and efficient carbohydrate active enzymes (CAZymes) that are capable of modifying or degrading plant-derived carbohydrates underpins the food and feed industries as well as renewables-based biotechnology. Of particular interest are enzymes capable of degrading complex lignocellulose, generating simple sugars from which biochemicals and second generation biofuels can be produced. This transformation would benefit from more efficient and cheaper enzyme mixtures, enabled by the discovery of enzymes with improved stability, novel or improved catalytic mechanisms or other helper proteins that contribute to synergistic substrate degradation^1^,^2^. Genome sequencing of many microbes with high lignocellulose degradative capacity has resulted in the discovery of many potential valuable CAZymes, (available in the CAZy database^3^), but their biochemical characterization is lagging behind^4^. Furthermore, detailed understanding of the regulation behind gene expression and protein secretion in fungal enzyme production strains could optimize the industrial enzyme production process^5^,^6^.

The fungus *Aspergillus niger* is extensively used as an industrial producer of organic acids and enzymes^7^. The genome of this fungus encodes a large set of CAZymes for the degradation of plant polysaccharides^8^, and these genes are expressed in response to cultivation on lignocellulosic substrates that are of relevance as feedstock for biofuel production, such as wheat straw, willow and sugar cane bagasse^9^,^10^,^11^. Despite the abundance of research on *A. niger* CAZymes, the biochemical characterisation of many of these enzymes is incomplete. This lack of knowledge on enzyme specificities, in particular substrate and product range, prevents a complete understanding of the enzymatic machinery responsible for the degradation of lignocellulose. Methods for rapid, sensitive detection and characterisation of enzyme activity on plant-derived substrates are therefore an essential tool. However, key challenges are posed by a trade-off between method throughput and derived information content, and the limited availability of characterised complex substrates.

Carbohydrate arrays are eminently suited to resolve the key challenges associated with characterising CAZyme activity, namely simultaneous screening of multiple reactions and conditions on minimal amounts of substrates. Carbohydrate arrays, including those carrying plant-based oligosaccharides^12^,^13^, have been successfully used to screen for enzyme activities as well as binding specificities of antibodies and carbohydrate binding proteins (for recent reviews see ref. 14, 15, 16). These arrays offer high analytical resolution as the complex structural features of the polysaccharides are separated in sub-structures. However, a limitation of many arrays is their reliance on labelled substrates, binding domains or antibodies to aid visualisation. The coupling of carbohydrate arrays with MALDI-ToF MS enables sensitive, rapid and label-free visualisation of enzymatic products directly at the array surface and can also allow for structural features to be ascertained through tandem MS^17^. Recently, carbohydrate arrays coupled with MS have been applied to determine the substrate specificity of carbohydrate binding modules and to detect and identify exo-glycosidase enzyme activities, active on terminal residues of the attached carbohydrates^13^,^14^, as well as limited endo-acting activity on short oligosaccharides^18^.

Here, we expanded on this work by generating MALDI-ToF MS compatible carbohydrate arrays with plant-derived oligosaccharides, including those with a high degree of polymerisation (DP), and applying them to detect and identify substrates and products of both endo- and exo- acting fungal CAZymes, including a proof of principle application towards detection of activity of lignocellulose-active CAZymes enzymes secreted by *A. niger*. From plant polysaccharides we produced, isolated and characterized oligosaccharides with a high DP. We use these, as well as commercially available oligosaccharides, to generate a carbohydrate array on a hydrophobic self-assembling monolayer (SAM) of alkanethiols coating the gold surface of a MALDI-ToF target (Fig. 1). We show that this system can be used to detect both endo- and exo-acting enzyme activity and we apply the arrays to detect substrate changes caused by activity of CAZymes of *A. niger*.

## Methods

### Fungal strains and growth conditions

The fungus *Aspergillus niger* strain AB4.1^19^ was grown on potato dextrose agar slopes at 28 °C until spores were produced. All AB4.1 cultures were supplemented with 10 mM uridine. Spores were harvested with 0.1% (v/v) Tween 20 and liquid cultures of 100 ml aspergillus minimal medium (AMM)^9^ with 1% glucose were inoculated with 10^6^ spores ml^−1^ and incubated at 28 °C, 150 RPM for 48 h. Mycelium was harvested using Miracloth, washed with AMM without carbon source, and 1.5 g (wet weight) was transferred to cultures of 100 ml AMM with 1% (w/v) ball milled wheat straw and incubated for 24 h at 28 °C, 150 rpm. Preparation and composition of the straw has been described previously^9^. Mycelium and culture filtrate were separated by filtration through Miracloth, the culture filtrate was concentrated 20-fold using Vivaspin 20 columns with a 10 kDa Mw cut-off and frozen until further analysis.

### Gene expression analysis

Fungal mycelium was disrupted by grinding it in liquid nitrogen using a mortar and pestle. RNA was isolated using Trizol (Invitrogen), followed by clean-up and DNAse treatment using the NucleoSpin RNA purification kit (Macherey-Nagel). Absence of genomic DNA was verified by PCR. cDNA was synthesized using SuperScript 3 reverse transcriptase (Invitrogen) using 0.5 μg total RNA and oligo(dT) primer. To measure expression of *cbhA, cbhB, xynB* and *actA,* qRT-PCR was performed on an Applied Biosystems 7500 Fast Real-Time PCR system in a 10 μl total volume with 2 μl 4× diluted cDNA, 0.2 μM of each primer (Supplementary Table S1) and 5 μl FAST SYBR-Green Master Mix (Applied Biosystems). qRT-PCRs were performed with a 95 °C 20 s initial denaturation, followed by 40 cycles of 95 °C for 3 s and 60 °C for 30 s, with all measurements in technical duplicate. Production of a single product was verified using a melt-curve. Gene expression levels were calculated from a genomic DNA standard curve, and corrected for expression of the reference gene^20^ *actA*. Values are given as mean ± standard error of biological triplicate experiments.

### Protein analysis

Proteins in fungal culture filtrate were separated on 4–20% Tris-glycine Novex SDS-PAGE gels (Invitrogen) and visualized with silver staining^21^. For protein identification, proteins were precipitated by adding 5 ml trichloroacetic acid to 20 ml culture filtrate, incubating for 1 h at 4 °C and spinning at 16060 *g* for 10 min. Pellets were washed twice with 200 μl acetone, loaded onto a 10% SDS-PAGE gel and electrophoresis performed until the dye front had moved approximately 1 cm into the separating gel. Each gel lane was then cut into a single slice, on which in-gel tryptic digestion was performed using a DigestPro automated digestion unit (Intavis Ltd).

Peptides were analysed generally as described by^22^ with the following exceptions: an Acclaim PepMap C18 nano-trap column (Thermo Scientific) was used for injection. Peptides were resolved with a 7 segment gradient (1–6% solvent B over 1 min, 6–15% B over 58 min, 15–32% B over 58 min, 32–40% B over 5 min, 40–90% B over 1 min, held at 90% B for 6 min and then reduced to 1% B over 1 min), Tandem mass spectra were acquired in the mass range m/z 300 to 2000, followed by MS/MS for the top twenty multiply charged ions. Data were processed with Proteome Discoverer software v1.4 (Thermo Scientific), searched against *the A. niger* SwissProt database (downloaded 23-03-16) using the SEQUEST algorithm, with a 10 ppm peptide precursor tolerance and a maximum of 1 missed cleavage, and peptide data was filtered to meet a false discovery rate of 1% while retaining only proteins identified by ≥2 unique peptides. The mass spectrometry proteomics data were deposited to the ProteomeXchange 7 Consortium via the PRIDE partner repository with the dataset identifier PXD005699 and 10.6019/PXD005699.

### Isolation of hemicellulose and production of oligosaccharides

The hemicellulose fraction of wheat straw was isolated generally as described elsewhere^23^. Briefly, 3% (w/v) wheat straw was suspended in 0.5 M KOH at 40 °C for 2.5 h, after which the suspension was filtered through Miracloth and neutralized with 6 M acetic acid. Polymers were precipitated by adding 3 volumes of ethanol and incubation at 4 °C for ≥1 h. Precipitate was collected by centrifugation, washed with 70% (v/v) ethanol and lyophilized, resulting in the recovery of 2.4 g hemicellulose enriched fraction from 16 g of wheat straw.

Oligosaccharides were generated by hydrolysis of 100 mg hemicellulose aliquots in 2 ml 0.5 M H_2_SO_4_ for 10 min at 100 °C, after which samples were cooled and neutralized with sodium carbonate. Water-soluble material was collected and lyophilized. The material (0.7 g) was dissolved in 10 mM ammonium bicarbonate, rid of particles by centrifugation, and separated on size on a Bio-Gel P4 fine (Biorad) column with a diameter of 0.9 cm and length of 70 cm. The column was equilibrated and run in 10 mM ammonium bicarbonate at 12 ml h^−1^ using a peristaltic pump, and elution fractions of 3 ml were collected for 5 hours.

Wheat arabinoxylan (Medium viscosity, Megazyme) was hydrolysed to generate oligosaccharides using *Thermomyces lanuginosus* endo-xylanase (X2753, Sigma Aldrich). A 1% (w/v) solution of arabinoxylan in boiling water was prepared, and cooled to 60 °C. Xylanase (8 mg ml^−1^ suspension in sodium citrate buffer pH 6) was added as 200 μl per g arabinoxylan and incubated for 30 min at 60 °C. The suspension was boiled for 5 min to stop enzyme activity, cooled and lyophilized. Recovered soluble material (1 g) was dissolved in 4 ml 10 mM ammonium bicarbonate and separated on the Bio-Gel column as described above.

### Characterization of oligosaccharides

Fractions containing oligosaccharides were analysed by TLC, MALDI-ToF-MS and NMR. TLC was performed by separating samples on TLC silica 60 plates with aluminium backing (Merck), and a liquid phase of butanol: ethanol: water in 5:5:3 ratio (v/v). Carbohydrates were visualized by dipping the plate in orcinol solution (250 mg orcinol (Sigma) in 95 ml ethanol, 5 ml H_2_SO_4_) and heating the plate to 100 °C. Samples were prepared for MALDI-ToF by co-crystalizing them with a 5% 2,5-dihydroxybenzoic acid (DHB) matrix solution in methanol. MALDI-ToF MS was performed using the Bruker Ultraflex 3 in positive mode.

Selected samples were exchanged with deuterium oxide and analysed by NMR. Measurements were made on a Bruker AV(III)500 NMR spectrometer using a dual ^1^H/^13^C helium-cooled cryoprobe operating with a sample temperature of 298 K. Spectra were approximately referenced in the ^1^H dimension using the deuterium lock (equivalent to setting the HDO peak = 4.75 ppm). Accurate referencing was achieved by setting the easily identifiable reducing end α-xylose resonance equal to 5.184 ppm. This procedure gives chemical shifts directly equivalent to those referenced relative to internal acetone at (δ = 2.225 ppm) as given in ref. 24. The ^13^C chemical shifts were measured relative to external DSS (4,4-dimethyl-4-silapentane-1-sulfonic acid) set to −1.6 ppm and thus are indicative only.

^1^H NMR spectra were recorded with 30 degree pulses, a data acquisition time of 3.17 s and a relaxation delay of 1 s. The spectral width was 20.6 ppm. Data were zero-filled and multiplied by an exponential window function with lb = 0.3 Hz prior to Fourier transformation. Spectra were baseline corrected and integrated using the standard routine in the Bruker TOPSPIN software (no lineshape fitting was attempted).

HSQC spectra were acquired phase sensitive in echo/anti-echo mode in a 1k × 256 data matrix using the Bruker pulse program hsqcetgpsisp2.2 which utilises double inept transfer. ^13^C decoupling was applied during acquisition. Prior to Fourier transformation, data points in the F1 dimension were doubled using linear prediction (64 coefficients), the data matrix was zero-filled to 2k × 1k real data points. A cosine-squared window function was applied to the data.

TOCSY spectra were acquired phase sensitive in echo/anti-echo mode in a 2k × 512 data matrix using the Bruker pulse program dipsi2etgpsi which utilises the DIPSI 2 sequence for mixing. The mixing time was set to 15 ms to observe direct correlations or 80 ms to observe coupling networks. Prior to Fourier transformation, data points in the F1 dimension were doubled using linear prediction, the data matrix was zero-filled to 4k × 1k real data points. A cosine-squared window function was applied to the data.

### Preparation of carbohydrate arrays

Maltose (Sigma-Aldrich), cellotetraose, cellohexaose and xylohexaose (Megazyme) were coupled to hexadecylamine via reductive amination; 1–5 mg carbohydrate with hexadecylamine (2: 1 molar ratio), 1 M sodium cyanoborohydride in 1 ml of 90% (v/v) DMSO, 10% (v/v) acetic acid and incubated overnight at 70 °C. Reactions were diluted with methanol before use.

Reductive amination of non-commercial oligosaccharides (hemicellulose fractions H5, H6, H7, H9 and arabinoxylan fractions AX10, AX11, AX12, AX15) was performed according to an alternative method based on the work of Gildersleeve *et al*.^25^. Briefly, oligosaccharides (0.4 mg) were dissolved in a mixture of sodium borate buffer (31 μl of a 400 mM solution, pH 8.5) and sodium sulfate buffer (21 μl of a 3 M solution, 50 °C), then hexadecylamine (12 μl from 6.43 mg of hexadecylamine in 3 ml of methanol) and sodium cyanoborohydride (1.77 mg) were added. The reaction was allowed to warm at 56 °C. Labelling of the oligosaccharides could be visualised by application of 1 μl of α-cyano-4-hydroxycinnamic acid (CHCA) (20 mg ml^−1^ in a mixture of 50% (v/v) acetonitrile and 50% (v/v) water containing 0.1% of trifluoroacetic acid (TFA)) followed by MALDI-ToF MS. After 8 h, the reaction mixture was cooled at room temperature, diluted in water/methanol (2 ml, typically in a 4:1 ratio) and stored at room temperature.

Gold-coated MALDI target plates (AB Sciex Ltd (AB plates)) were cleaned with a mixture of 30% (v/v) hydrogen peroxide and 70% (v/v) sulphuric acid for 20 min, rinsed extensively with water, followed by methanol and dried under a stream of nitrogen. Then, the plates were incubated overnight in 1-undecanethiol (37.5 μl in 20 mL of methanol) forming a hydrophobic SAM. Plates were washed with methanol, dried under a stream of nitrogen, and 1 μl of hexadecylamine labelled oligosaccharides, diluted to 0.2 mg ml^−1^ in water:methanol (4:1, vol/vol), were spotted on each well. After incubation for 20 min in a sealed container, plates were washed twice with water. MALDI-ToF MS confirmed immobilization after application of 1 μL of DHB (15 mg ml^−1^ in a mixture of 50% (v/v) acetonitrile and 50% (v/v) water containing 0.1% of TFA) directly on the gold plate.

### Enzyme reactions on carbohydrate arrays

Reaction conditions for commercial enzymes were designed for incomplete substrate degradation, thus allowing identification of both substrates and products in one mass spectrum. β-glucosidase from almond (49290, Sigma-Aldrich) was prepared as 1 mg ml^−1^ (≥6 U ml^−1^) in water, then 2 μl was spotted on the carbohydrate array and the plate was incubated for 30 min at 37 °C in a sealed container. Endo-xylanase from *Thermomyces lanuginosus* (X2753, Sigma Aldrich) was prepared as 5 ng ml^−1^ (≥10 mU ml^−1^), mixed 1:1 with 100 mM citric acid-sodium phosphate buffer (pH 8), then 2 μl was spotted on the carbohydrate array and the plate was incubated for 10 min at 30 °C in a sealed container. Measurements to determine the minimal enzyme amount that can reliably be detected were performed with a dilution range of xylanase, incubated at pH6 for 2 h at 30 °C. Culture filtrates from *A. niger* grown on wheat straw (1-fold or 20-fold concentrated) were mixed 1:1 with 100 mM citric acid-sodium phosphate buffer (pH4), then 2 μl were spotted on the carbohydrate array and the plate was incubated for 2 h at 30 °C in a sealed container. After incubation, the plates were dipped into distilled water and shaken gently for 1 min, and then dried under a stream of nitrogen. Reaction products were identified with MALDI-ToF MS and MS-MS on a Ultraflex II TOF/TOF, using 2′, 4′, 6′-trihydroxyacetophenone monohydrate (THAP) (10 mg ml^−1^ in acetone) or DHB (15 mg ml^−1^ in a mixture of 50% (v/v) acetonitrile and 50% (v/v) water containing 0.1% of TFA) as matrix.

### Mass spectrometric analysis of arrays

Gold AB plates were loaded into the instrument using the MTB AB adapter (Bruker). MALDI-ToF mass spectra (1500 shots/spectrum) were recorded on a Bruker Ultraflex II instrument with a Smartbeam I laser in positive reflector ion mode. The instrument was calibrated between *m/z* 700–3500 using a solution of peptide calibration mix II (Bruker Daltonics, Bremen). Data were analysed and normalised using FlexAnalysis version 3.0 (Bruker).

## Results

### Hydrolytic enzymes secreted by *A. niger* grown on wheat straw

**Figure 2:**
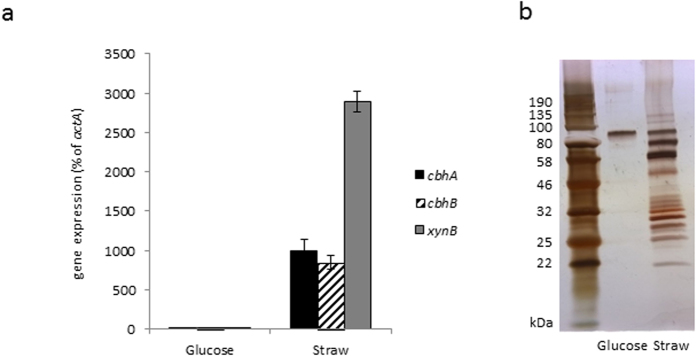
Analysis of gene expression and protein secreted by *A. niger*. (**a**) Expression of genes *cbhA* (encoding cellobiohydrolase CbhA), *cbhB* (encoding cellobiohydrolase CbhB), and *xynB* (encoding xylanase XynB), as % of expression of actin encoding gene *actA*, in liquid cultures containing glucose or wheat straw as carbon source, represented as mean ± standard error, n = 3, (**b**) SDS-PAGE gel of proteins secreted by *A. niger* in these cultures.

The fungus *A. niger* produces a range of plant polysaccharide degradative enzymes in response to lignocellulose. The fungus was cultivated in liquid cultures containing glucose in order to obtain biomass, and resulting mycelium was washed and transferred for 24 h to liquid cultures containing ball milled wheat straw. These cultivation conditions have previously been found to highly induce a large number of genes that encode (putative) plant polysaccharide active enzymes^9^. qRT-PCR showed that such genes were induced as transcript levels of the cellobiohydrolases encoding genes *cbhA, cbhB* and the xylanase encoding gene *xynB* were strongly increased compared to the repressive glucose conditions (Fig. 2a). Culture filtrate was harvested and analysis using SDS-PAGE; a broad range of proteins are indeed secreted in wheat straw cultures (Fig. 2b). Shotgun proteomics of precipitated proteins from the wheat straw culture filtrate (Supplementary Table S2) shows that the most abundant proteins are annotated as (putative) CAZymes active on cellulose and hemicelluloses, in particular (arabino)xylan, and their predicted activities constitute a mixture of exo- and endo-acting enzymes. Cellulolytic enzymes include (putative) cellobiohydrolases CbhA (GH7) and CbhB (GH7), CbhC (GH6), endoglucanase EglB (GH5-5), and several putative β-glucosidases. Xylanolytic enzymes include (putative) endoxylanases XynA (GH10) and XynB (GH11), β-xylosidase xlnD (GH3), α-arabinofuranosidases AxhA (GH62), AbfB (GH54), AfbA (GH51). Based on these results, cellulose oligosaccharides as well as (arabino)xylan-derived oligosaccharides were prioritized as substrates for the carbohydrate arrays.

### Production of oligosaccharides

To obtain broad structural variety, (arabino)xylan oligosaccharides were generated from multiple sources. The xylan-rich hemicellulose fraction isolated from wheat straw was digested using mild acid hydrolysis, whereas commercially obtained wheat arabinoxylan was enzymatically hydrolysed. The obtained oligosaccharide mixtures (referred to as fractions Hn and AXn respectively, where n indicates the fraction number) were subjected to size exclusion chromatography to remove monomers and low molecular weight oligosaccharides, as well as the remaining polysaccharide. Separation and visualisation of the soluble hemicellulose-derived hydrolysis products (H) on TLC, (Supplementary Fig. S1a), showed a range of oligosaccharides as well as monomers. MALDI-ToF MS indicated a series of pentose-oligosaccharides with a degree of polymerisation (DP) of up to at least 15 (Supplementary Fig. S1b). TLC analysis of arabinoxylan derived oligosaccharides (AX)(Supplementary Fig. S1c) showed generation of an oligosaccharide series, and MALDI-ToF MS identified masses consistent with pentose oligosaccharides with a range of DPs from 5 to well over 20 (Supplementary Fig. S1d). Oligosaccharide fractions were selected that contained mainly oligosaccharides with a DP of 5–15 (fractions H5-H9) and 5–20 (fractions AX10-AX15).

**Figure 3:**
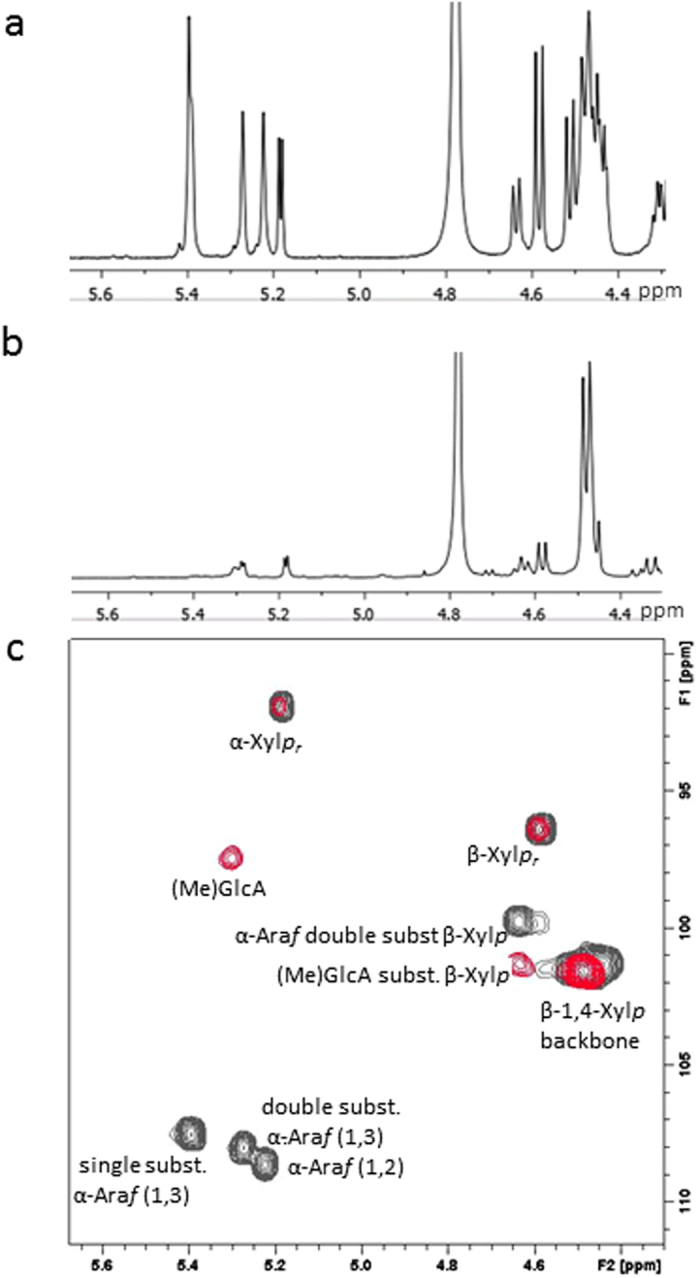
NMR analysis of oligosaccharides. (**a**) 1D ^1^H spectrum of arabinoxylan fraction AX14, (**b**) 1D ^1^H spectrum of wheat hemicellulose fraction H8 and (**c**) HSQC spectrum showing the anomeric reporter resonances of fraction H8 (red), overlaid with that of fraction AX14 (black). The assigned carbohydrate monomers and linkage types are indicated.

The identity and structure of obtained oligosaccharides was further analysed with NMR. Analysis of fraction H8 showed that it contained, as main component, a linear β-(1,4)-Xyl*p* oligosaccharides with an average DP of ~7, as derived from chemical shifts observed in the ^1^H−^1^H TOCSY NMR spectrum of fraction H8 (Supplementary Table S3a). The 1D ^1^H NMR and 2D ^1^H-^13^C HSQC NMR chemical shifts (Supplementary Table S3b) of fractions H6, H7, H8 also corresponded with oligosaccharides with a linear β-1,4-Xyl*p* backbone. Based on chemical shifts reported for similar structures^26^, the oligosaccharides contained 4-*O*-methyl-glucuronic acid (MeGlcA*p*) and glucuronic acid (GlcA*p*) decorations (Fig. 3). The H-fractions varied in backbone DP and number of decorations. Integration of the 1D ^1^H NMR signals (Supplementary Table S3c) indicated that the oligosaccharides have an average backbone DP of 10.4, 8.3 and 7.9 for H6, H7 and H8 respectively, with an average of 1.1, 0.8 and 0.6 decorations per oligosaccharide.

1D ^1^H NMR and 2D ^1^H-^13^C HSQC NMR chemical shifts of fractions AX14 and AX15, reported in Supplementary Table S3b, indicated that these samples also contain oligosaccharides with a linear β-1,4-Xyl*p* backbone (Fig. 3). No (Me)GlcA*p* decorations were observed, but the oligosaccharides contained one or more xylose residues with a single or double α-Ara*f* substitutions, as indicated by comparison of chemical shifts with those reported for similar structures^27^. Integration of the 1D ^1^H NMR signals indicated that the oligosaccharides have an average xylose backbone DP of 4.9 and 4.3 for AX14 and AX15 respectively. The ratio of oligosaccharides with single and double α-Ara*f* substitutions differed slightly between fraction AX14 and AX15. On average, AX14 had 0.9 single or 0.4 double Ara*f* substituted xylose residues respectively and AX15 had 0.7 single or 0.3 double Ara*f* substituted xylose residues.

### Carbohydrate array production

A hydrophobic self-assembling monolayer (SAM) on a gold plate was used as a scaffold for the immobilization of various labelled carbohydrates via hydrophobic interaction. A small library of oligosaccharides activated for attachment to the hydrophobic SAM was generated, by linking glycans covalently to a hydrophobic hexadecylamine tail using reductive amination. Using a classical method with 90% (v/v) DMSO, 10% (v/v) acetic acid^28^, commercially obtained cellotetraose, cellohexaose and xylohexaose were readily attached to hexadecylamine. However, as this procedure failed with isolated oligosaccharides from fraction AX (AX10, AX11, AX12, AX15) or fraction H (H5, H6, H7, H9), we developed an alternative method, largely inspired by the work of Gildersleeve *et al*.^25^, that successfully labelled oligosaccharides, as identified with MALDI-ToF MS. The unlabelled oligosaccharides were observed using DHB or THAP as matrix, while the labelled oligosaccharides can be analysed solely with CHCA. Carbohydrates labelled with hexadecylamine were immobilized on a gold plate that was functionalized with a hydrophobic SAM of 1-undecanethiol. After incubation, the plate was washed with water to remove non-immobilised carbohydrates, as well as the NaBH_3_CN from the reductive amination step, which may interfere with MS measurements and enzymatic activity.

All DP oligosaccharide fragments were retained upon labelling of AX and H fractions (Supplementary Fig. S2). Some discrepancies in relative intensity are observed between unlabelled and labelled (for example DP6 is relatively more intense in the labelled fraction AX10 (Supplementary Fig. S2a)). This may be the result of alterations in the ionisation efficiency of the two systems, differences in solubility as well as preferential labelling of certain oligosaccharides depending on their degree of polymerization and the presence of substitutions^25^,^29^,^30^,^31^,^32^. The signal intensity ratios between peaks during reductive amination reactions with an excess of hexadecylamine (Supplementary Table S4, procedure *a* vs *b*), indicated that preferential labelling contributed to the observed effect, as over time low DP oligosaccharides were initially labelled while the final signal intensity ratios were more in agreement with the unlabelled oligosaccharides. Influence of degradation of high DP oligosaccharides was excluded as no increase in signals corresponding to DP1-4 are observed after labelling and monitoring of the reductive amination reaction of fraction AX10 for up to 3 days showed that the signal intensity ratios between peaks were stable from 4 h up 3 days (Supplementary Table S4 procedure *a*). Conveniently, after immobilization onto the SAM, both low and high DP labelled oligosaccharides can still be measured by MS, albeit for high DP oligosaccharides with a much reduced signal (Supplementary Fig. S2). This may be a result of a reduced ionisation efficiency for these non-covalently immobilised systems, or preferential attachment of low DP oligosaccharides. However, we show here that higher DP oligosaccharides are successfully immobilized on this array platform, with products of well over DP10 detected in H9 and AX10 fractions with considerable signal intensity. Furthermore, comparison of peak ratios of oligosaccharides on arrays generated on separate days showed that interday reproducibility was excellent, with a variation in peak ratios below 4% relative standard deviation (Supplementary Table S5).

To estimate the surface capacity to bind labelled oligosaccharide and the effect of oligosaccharide concentration, oligosaccharides were applied on the plate in a range of concentrations from 3.1 mg ml^−1^ to 0.2 mg ml^−1^ in water:methanol (4:1, v/v). Regardless of the dilution, the spectra obtained were similar in all respects, suggesting that a 0.2 mg ml^−1^ concentration is sufficient for efficient reproducible array formation. This allows for 5000 assays per mg carbohydrate and equals application of 0.1–0.2 nmol carbohydrate.

The successful attachment of a range of commercial oligosaccharides as well as oligosaccharides in AX- and H-fractions shows that not only oligosaccharides with a low DP can be attached, but also linear and decorated oligosaccharides of considerably higher DP. Such longer oligosaccharides (mainly DP7-DP10 range) are rarely available commercially, and often not tested for carbohydrate array platforms^18^,^33^. However, these substrates are essential for adequate detection and characterisation of endo-acting enzymes since these enzymes often have extensive substrate binding clefts in which multiple binding sites contribute to substrate and product specificity^34^.

### Carbohydrate arrays are suitable to detect enzyme activity

In order to establish whether these carbohydrate arrays can be used as a method for the detection and identification of products resulting from enzyme activity, carbohydrate arrays were incubated with exo- and endo-acting enzymes and, after washing to remove sample and non-attached degradation products, reaction products were identified by MALDI-ToF MS.

**Figure 4:**
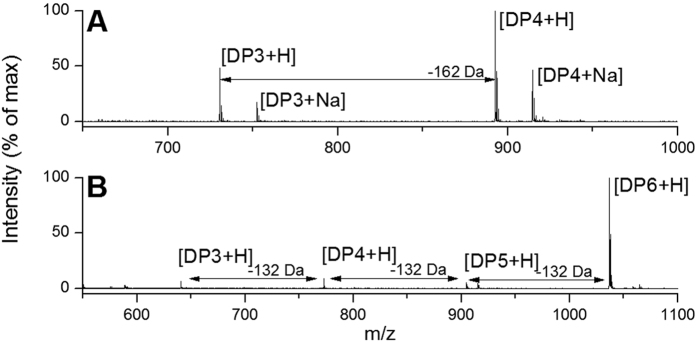
MALDI-ToF MS analysis of commercial enzyme activity on carbohydrate arrays. Spectra showing reaction products of incubation of (**A**) β-glucosidase on cellotetraose-containing arrays, with DP4 + Na 914.5 m/z, DP4 + H 892.5 m/z, DP3 + Na 752.4 m/z, DP3 + H 730.5 m/z, (**B**) endoxylanase on xylohexaose-containing arrays with DP6 + H 1036.7 m/z, DP5 + H 904.6 m/z, DP4 + H 772.6 m/z, DP3 + H 640.6 m/z. Reaction conditions were chosen such that incomplete substrate degradation allowed identification of both substrates and products in one mass spectrum. Intensities of peak resulting from background substrate degradation or impurities were ≤2.2% (Fig. S3).

Exo-acting activity of β-glucosidase cleaves terminal glucose residues from cellulose oligosaccharides. Incubation of β-glucosidase on cellotetraose on carbohydrate arrays resulted, as expected, in a reaction product with masses corresponding to hexadecylamide-labeled cellotriose (Fig. 4A). Endoxylanase from *Thermomyces lanuginosus* acting on xylohexaose containing carbohydrate arrays resulted in products with masses corresponding to hexadecylamide labelled xylopentaose, xylotetraose and xylotriose (Fig. 4B). The minimum xylanase enzyme concentration resulting in activity that could reliably be detected on the arrays with the xylohexaose substrate was 2.5 ng μl^−1^, equivalent to application of ≥5 μU of activity per reaction.

The identification of these reaction products shows that the application of carbohydrate arrays that display enzyme substrates, combined with label free detection by MALDI-ToF MS, is suitable for the detection of CAZyme reaction products and that both activity resulting from endo- and exo-acting enzymes can be detected.

### Carbohydrate arrays are suitable to detect enzyme activity in biological samples

Culture filtrates of the *A. niger* grown on wheat straw are complex with regard to composition, they contain proteins, organic acids, carbohydrates remaining from the lignocellulose substrate as well as unknown components including fungal metabolites. To test the suitability of carbohydrate arrays to identify products from enzyme activity in these samples, culture filtrates were incubated with carbohydrate arrays containing commercial xylohexaose and cellotetraose, as well as the arabinoxylan and hemicellulose derived oligosaccharides.

**Figure 5:**
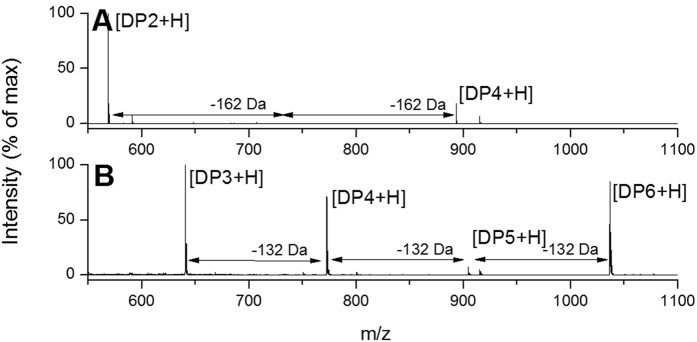
MALDI-ToF MS analysis of *A. niger* enzyme activity on carbohydrate arrays. Spectra showing reaction products of incubation of enzymes from *A. niger* wheat straw culture filtrate on (**A**) cellotetraose-containing arrays, with DP4 + H 893.1 m/z, DP2 + H 568.8 m/z, (**B**) xylohexaose-containing arrays, with DP6 + H 1036.5 m/z, DP5 + H 904.6 m/z, DP4 + H 772.5 m/z, DP3 + H 640.5 m/z.

Wheat straw culture filtrate incubated on cellotetraose containing arrays resulted in the generation of a masses corresponding to hexadecylamide-labeled cellobiose (Fig. 5A). These results are indicative of activity of non-reducing end acting cellobiohydrolases, which cleave cellobiose from the non-reducing substrate end, as well as possible β-glucosidase or endo-cellulase activity. These enzymes were also identified by proteomics to be present in the culture filtrate as highly abundant proteins. Incubation of wheat straw culture filtrate on immobilised xylohexaose resulted mainly in products with masses corresponding to labelled xylotetraose and xylotriose, with minor amounts of xylopentaose (Fig. 5B). MS-MS confirmed the identity of these products. These results are indicative of activity of enzyme(s) that sequentially cleaves xylose, preferentially cleaves either xylobiose or xylotriose from the non-reducing end of the substrate, or by random hydrolysis of the substrate. Based on the proteomics results, these activities are likely displayed by XynA, XynB and/or XlnD that were identified in the culture filtrate; these enzymes have endo-xylanase and β-xylosidase activities^35^,^36^,^37^.

**Figure 6:**
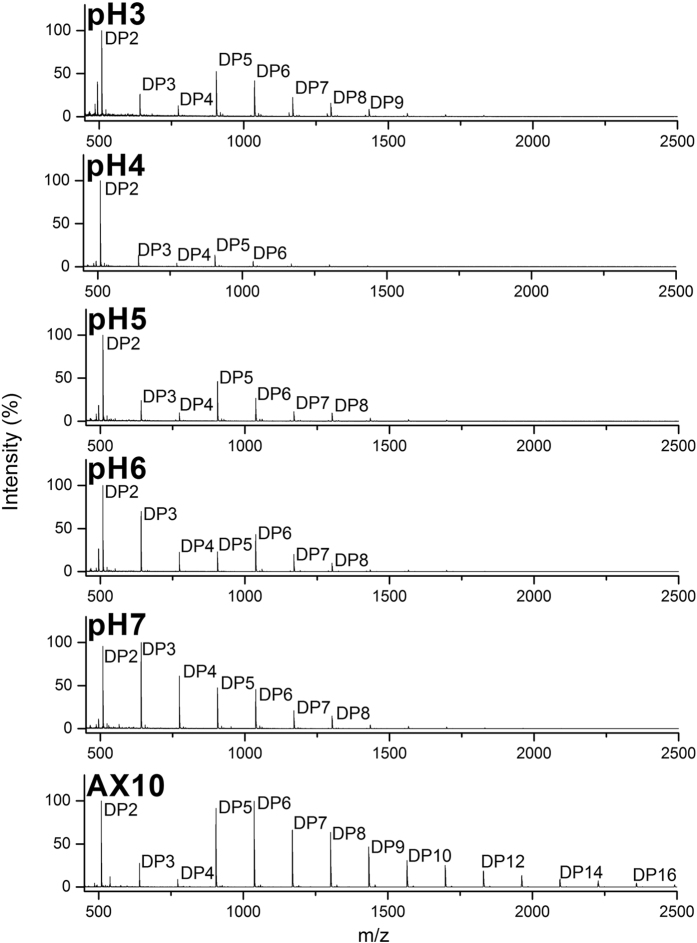
MALDI-ToF MS analysis of effect of pH on activity of *A. niger* enzymes on fraction AX10. Maldi-ToF MS spectra before and after incubation with enzymes from *A. niger* wheat straw culture filtrate on carbohydrate arrays containing fraction AX10, at 30 °C for 4 h using various pH conditions (from pH3 to pH7).

**Figure 7:**
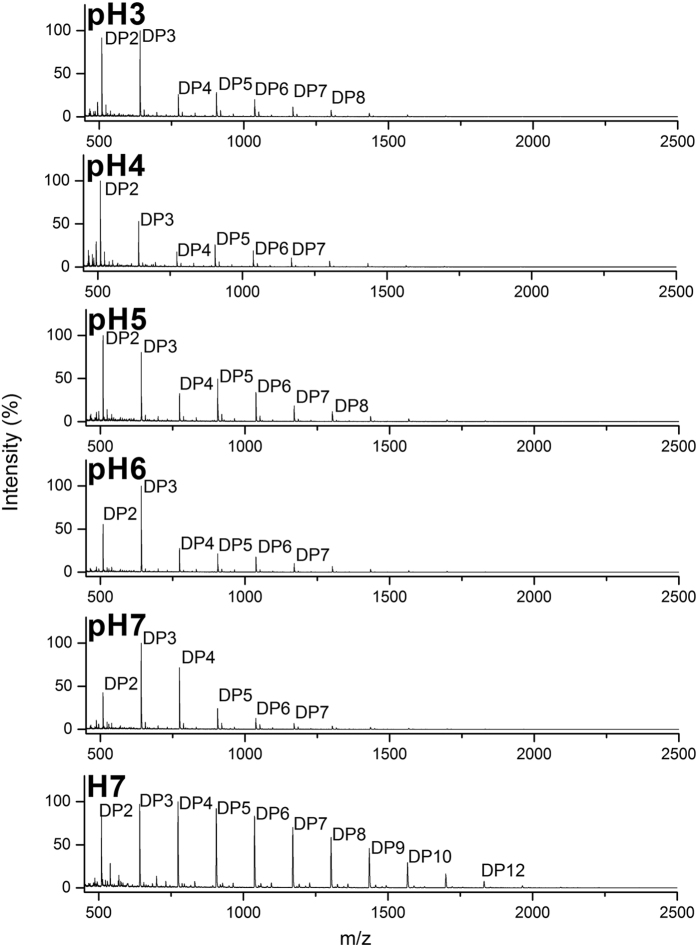
MALDI-ToF MS analysis of effect of pH on activity of *A. niger* enzymes on fraction H7. Maldi-ToF MS spectra before and after incubation with enzymes from *A. niger* wheat straw culture filtrate on carbohydrate arrays containing fraction H7, at 30 °C for 4 h using various pH conditions (from pH3 to pH7).

We studied the enzyme activity of a culture filtrates of *A. niger* on the high DP oligosaccharides of the AX and H fractions under several parameters including the reaction time (30 min, 2 h or 4 h) and pH (range from pH3 to pH7). On both AX and H fractions, enzyme activity in the wheat straw culture filtrates resulted in increased signal intensities corresponding to DP2, DP3 and often also DP4, while peaks from DP5 up to DP11 decreased, and those higher than DP12 were not identified (Figs 6 and 7). Activity could be expressed using the signal intensity ratio DP2/DP5, DP3/DP5 and, to a lesser extent, DP2/DP3.

During incubation of the culture filtrates of *A. niger* at pH4 and 30 °C, variation of the time of incubation from 0 h to 2 h to 4 h indicated that the longer reaction time decreased the amount of DP5 significantly, and increased the ratios DP2/DP5 and DP3/DP5 from 1.1 to 2.7 to 7.7 (±0.0) and from 0.3 to 0.7 to 1.0 (±0.1), respectively for AX10. These ratios increased for H7 from 0.9 to 1.7 to 4.3 (±0.1) and from 1 to 1.8 to 2.2 (±0.2). Longer incubation did not enhance the activity of the enzymes as the same ratios were obtained after 4 h and 8 h (data not shown).

The effect of the pH on wheat straw culture filtrate enzyme activity on the oligosaccharides of fractions AX and H fractions was tested in incubations at 30 °C for 4 h. For fraction AX10 (Fig. 6), degradation of high DP oligosaccharides (DP4 upward) was observed at all pH values, and main products were DP2 and DP3. The ratios DP2/DP5 and DP2/DP3 were found highest at pH4 (6.2 and 5.8 respectively) (Table 1) and lowest at pH7. Interestingly, the signal corresponding to DP3 and DP4 was found to be most abundant at pH6 and pH7, indicating accumulation of these oligosaccharides under these conditions.

Regarding activity of the enzymes on fraction H7 (Fig. 7), the DP2 to DP3 product ratio was >1 solely at pH4 and pH5 (Table 1). In addition, as described for the fraction AX10, the DP2 to DP3 ratio was lower at pH6 and pH7 (0.5) than for the starting oligosaccharide (0.8), reflecting an accumulation of trisaccharide.

*A. niger* seemed able to degrade the higher DP oligosaccharides and produce low DP oligosaccharides both in acid and in neutral condition (from pH3 to pH7) in both AX and H oligosaccharide fractions. However, pH dependent activity was observed since the enzyme mixture was less active at pH6–7, resulting in an accumulation of trisaccharide (DP3) and tetrasaccharide (DP4). These results have clearly indicated that the overall optimum pH for this mixture of enzymes on these substrates, using our platform, was at pH4.

## Discussion

Through this study, we have confirmed that the carbohydrate microarray-based technology combined with MALDI-ToF MS is a powerful strategy for the assessment of enzyme activity in many simultaneous experiments using small amounts of valuable carbohydrates. Linear as well as decorated oligosaccharides, with both low and high DP, can be efficiently immobilised on the arrays, after which their enzymatic degradation can be monitored by MALDI-ToF MS.

Both covalent and non-covalent attachment of carbohydrates on arrays has been described^15^,^16^,^17^. Some of these platforms require chemical modification via several synthetic steps to generate carbohydrates suitable for attachment, which is readily performed with monomers but less straight forward for oligosaccharides. To enable the generation of arrays with a range of oligosaccharides, which are generally available in small quantities, we selected an attachment strategy that requires minimal synthetic modification; the attachment of a hydrophobic anchor via reductive amination, which can immobilise the attached carbohydrate on a hydrophobic SAM. A relatively short hydrophobic anchor (C12) immobilised poorly in our hands during initial test, while longer (C18, double C16) anchors, as described^18^, provided effective immobilisation, resulting in the employment of the C16 hexadecylamine immobilisation anchor described here.

Reductive amination of oligosaccharide has been widely used in the past for several applications including conjugation of carbohydrate to proteins or other scaffolds in order to produce molecules of pharmaceutical utility, as well as derivatization of biological sugars affording better detection and analysis by liquid chromatography and/or mass spectrometry^38^. The procedure generally employed for reductive amination of free oligosaccharide was described by Roy *et al*.^39^ in 1984, using sugar in the presence of NaBH_3_CN and aqueous sodium borate buffer for 24 h at 37–50 °C. The alternative protocol developed by the group of Gildersleeve^25^ is rather similar, except for the use of salt additives such as sodium sulfate. The authors have suggested that high salt concentrations could make the removal of water molecule easier, and hence, might favour formation of the imine. We explored the role of the sodium sulfate buffer by performing reductive amination with or without this salt additives; the presence of sodium sulfate buffer accelerates the rate of the reaction.

Purification of labelled oligosaccharides, which is time consuming and leads to a loss of material, can be avoidable following our approach. As an example, with solely 0.4 mg of oligosaccharide starting material, we could perform up to 2000 array experiments. However, without such purification no estimate can be obtained of the yield of the labelling reaction, making it impossible to compare yield between different oligosaccharide fractions. However, by comparing the signal intensity obtained during the MS analysis for each compound in the AX and H fractions over time, we showed that oligosaccharides with a low DP were labelled more effectively than those with the highest DP, which seems to be remedied by addition of amine.

The identification of reaction products from degradation of plant-biomass derived oligosaccharides in complex samples as these fungal culture filtrates, as well of that of commercial enzyme preparations, shows that the hydrophobic carbohydrate array coupled with MS was a platform that was well suited for measurement of such enzyme activities. The enzyme activities that were detected using the linear commercial oligosaccharides confirmed that the platform is suitable for detection of both exo- and endo-acting enzymes. Analysing enzyme activity on a single substrate, as performed here, gives a good indication of the presence or absence of degradative activities. It however has limited value in deriving a mechanism of action for an enzyme because the activity of a single enzyme during substrate degradation can result in multiple products: to study the substrate/product profile of a single enzyme in detail, separate incubations of the enzyme with a set of defined substrates of different lengths will be required.

For example, β-glucosidase hydrolyses the terminal residue of cellulose oligosaccharides, and degradation of substrate cellotetraose can thus theoretically result in cellotriose, and after another hydrolysis event on the same molecule, also in cellobiose. We did not observe the formation of cellobiose under our reaction conditions, which – besides steric effects - may point towards cellotetraose being the minimum length substrate for this particular enzyme, or, more likely may reflect a higher turnover rate for cellotetraose vs cellotriose. Similarly, different fits of substrates in the active cleft of the endo-acting enzyme may result in a range of different products, which may or may not be degraded further dependent on the minimum substrate length required by the enzyme and any differences in kinetics that it displays for these different substrates. This explains the range of products resulting from endo-xylanase activity observed in this study.

Using mixtures of such plant derived oligosaccharides with high DP, *i.e*. AX and H fractions we demonstrated that the optimum conditions required to observe an activity of the culture filtrates of *A. niger* on arabinoxylan or xylan oligosaccharides was an incubation at pH4 for 4 h at 30 °C. At higher pH, accumulation of DP3 and DP4 oligosaccharides was observed. For the oligosaccharides from the AX fractions, this could be due to reduced activity of α-arabinofuranosidases such as AbfB and AxhA that removes the arabinose decorations from the xylose backbone. However, since the same phenomenon was also observed for the H-fraction oligosaccharides, it is more likely due to a reduction in an exo-acting β-xylosidase that degrades the xylose backbone of DP4 and DP3 to DP2. The proteomics results indicated that such an enzyme, XlnD, was indeed present in the culture filtrates of *A. niger* grown on wheat straw.

Some divergence existed of the activity of *A. niger* enzymes in the culture filtrate when degrading oligosaccharides in the H7 and AX10 fractions; the latter seems to be a better substrate for the enzyme mixture as the reduction in maximum observed DP was much greater for AX (from DP20 to DP9) than for H (from DP14 to DP11). It is surprising that the AX fraction seems to be degraded more efficiently, since the structure is more complex (i.e. decorated with arabinose) compared to H (linear xylose series). However, the complex structure also means that a higher number of enzymes that are all highly abundant present in the culture filtrate can act on the oligosaccharide; not only the XynA GH10 and XynB GH11 endo-xylanases but also the arabinofuranosidases AfbB and AxhA. Furthermore, GH10 xylanases as XynA - which is the most abundant protein identified in the culture filtrate - are reported to more efficiently degrade arabinose-decorated xylose oligosaccharide compared to undecorated oligosaccharides^34^, thus possibly contributing to quicker turnover of AX than H oligosaccharide series. Alternatively, steric interference may contribute to the observed differences in degradation between the H and AX fractions, as close proximity of substrate molecules on the SAM may limit their availability to enzymatic modification or degradation. This underlines the need to immobilise a minimal amount of substrate on the SAM to limit such interferences.

No signal corresponding to DP1 was observed in all the conditions assayed, probably because the reductive amination yielded an unnatural ring-opened product, which could be not recognized as a substrate by the enzyme and led to an accumulation of DP2. This also suggested that *A. niger* enzymes need a closed-ring hemiacetal monosaccharide as backbone in order to be active.

In conclusion, we reported on the production of a carbohydrate array with plant polymer-derived oligosaccharides, and its application to detect activity of carbohydrate active enzymes in commercial preparations as well as biological samples. To our knowledge, this is the first report describing the use of a carbohydrate array with high DP oligosaccharide substrates in combination with MALDI-ToF MS. By using mass spectrometry instead of labelled antibodies in combination with the arrays, we made a significant advancement in detection of the substrate alterations that result from enzyme activities. This opens up the possibility of distinguishing different enzyme activities, such as exo- or endo-active enzymes, based on their product profiles. It also offers the opportunity to detect and distinguish between enzyme activities with different mechanisms to hydrolyse the substrate, such as LMPOs, lyases and endo-acting hydrolyses, as well as potential new mechanisms. This platform could form a basis for on-chip identification of complex enzyme products in combination with a recently developed carbohydrate sequencing method based on ion mobility-mass spectrometry (IM-MS)^39^,^40^,^41^,^42^.

## Additional Information

**How to cite this article**: van Munster, J. M. *et al*. Application of carbohydrate arrays coupled with mass spectrometry to detect activity of plant-polysaccharide degradative enzymes from the fungus *Aspergillus niger. Sci. Rep.* **7**, 43117; doi: 10.1038/srep43117 (2017).

**Publisher's note:** Springer Nature remains neutral with regard to jurisdictional claims in published maps and institutional affiliations.

## Supplementary Material

Supplementary Files

## Figures and Tables

**Figure 1:**
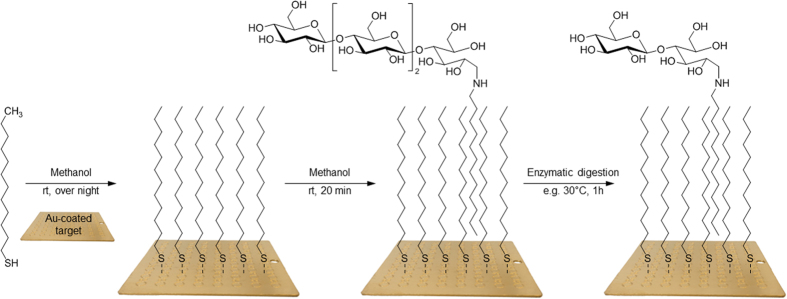
Schematic overview of carbohydrate array construction and use. After formation of a hydrophobic SAM of alkanethiols on a gold-coated target, hexadecylamine-labelled oligosaccharides are immobilised via hydrophobic-interaction. Products of enzyme activity, here exemplified by removal of DP2, as well as the original substrate, can be identified with MALDI-ToF MS.

**Table 1:** Ratio of the intensities of signal calculated for DP2/DP5, DP3/DP5, DP2/DP3 concerning fractions AX10 and H7 after incubation with *A. niger* enzymes at 30 °C for 4 h using various pH conditions (from pH3 to pH7).

	DP2/DP5		DP3/DP5		DP2/DP3	
	*AX10*	*H7*	*AX10*	*H7*	*AX10*	*H7*
pH3	2.2 ± 0.1	3.2 ± 0.0	0.5 ± 0.0	3.4 ± 0.3	4.3 ± 0.1	0.9 ± 0.0
pH4	7.7 ± 0.0	4.4 ± 0.1	1.0 ± 0.1	2.4 ± 0.2	7.8 ± 0.1	1.8 ± 0.1
pH5	2.2 ± 0.1	2.1 ± 0.0	0.5 ± 0.0	1.7 ± 0.1	4.3 ± 0.1	1.2 ± 0.1
pH6	4.6 ± 0.1	2.7 ± 0.0	1.4 ± 0.1	4.9 ± 0.0	3.2 ± 0.1	0.5 ± 0.0
pH7	2.1 ± 0.1	1.9 ± 0.1	2.1 ± 0.0	4.2 ± 0.1	1.1 ± 0.1	0.5 ± 0.1
No enzyme (starting material)	1.1 ± 0.0	0.7 ± 0.3	0.3 ± 0.0	0.8 ± 0.2	3.4 ± 0.1	0.8 ± 0.1

Values are given as mean ± standard deviation, n = 2.
