# Supplementary material for: Application of carbohydrate arrays coupled with mass spectrometry to detect activity of plant-polysaccharide degradative enzymes from the fungus Aspergillus niger
Source: Sci Rep. 2017 Feb 21;7:43117. doi: 10.1038/srep43117 (PMC5318901; doi:10.1038/srep43117)
Supplement: Supplementary Files [file srep43117-s1.pdf]

Application of carbohydrate arrays coupled with mass spectrometry to detect activity of plant-polysaccharide degradative enzymes from the fungus *Aspergillus niger*

Jolanda M van Munster[1], Baptiste Thomas[2], Michel Riese[2], Adrienne L Davis[3], Christopher J Gray[2], David B Archer[1], Sabine L Flitsch[2]

[1] Fungal Biology and Genetics, School of Life Sciences, University of Nottingham, University Park, Nottingham NG7 2RD, UK

[2] Chemical biology, Manchester Institute for Biotechnology, University of Manchester, 131 Princess Street, Manchester M1 7DN, United Kingdom

[3] School of Chemistry, University of Nottingham, University Park, Nottingham NG7 2RD, UK

Supplementary information

Supplementary Information:

Supplementary Figure S1: Thin layer chromatography of **a** oligosaccharides generated by acid hydrolysis of wheat hemicelluloses and **b** of oligosaccharides generated by enzymatic hydrolysis of wheat arabinoxylan, with relevant fractions labelled below the sample application site (numbers H1-H11 and AX5-AX18) and M indicating the H and AX mixture of oligosaccharides before size separation, **c** MALDI-ToF MS spectra with indicated the DP (DPx) and spacing of the main pentose oligosaccharide series and spacing of a minor secondary series consisting of 4Me-GLcA decorated xylose oligosaccharides for oligosaccharides generated by acid hydrolysis of wheat hemicelluloses and **d** for oligosaccharides generated by enzymatic hydrolysis of wheat arabinoxylan. All labelled peaks are sodium adducts.

Supplementary Figure S2. a MALDI-ToF MS spectra for fraction AX10 with (a1) unlabeled starting oligosaccharide, (a2) labelling of free oligosaccharide by reductive amination and (a3) immobilization of labelled carbohydrates on the gold plate via hydrophobic interactions **b** MALDI-ToF MS spectra for fraction H9 with (b1) unlabeled starting oligosaccharide, (b2) labelling of free oligosaccharide via reductive amination and (b3) immobilization of labelled carbohydrates on the gold plate via hydrophobic interactions.

Supplementary Figure S3. MALDI-ToF MS analysis of signals derived from background hydrolysis and/or impurities in carbohydrate arrays used with commercial enzyme activity. Spectra showing substrate incubated with buffer only under reaction conditions of **a** β-glucosidase on cellotetraose-containing arrays, with DP4 + Na 914.5 m/z, DP4 + H 892.5 m/z (100%), DP3 + Na 752.4 m/z, DP3 + H 730.5 m/z (2.2%) while DP3 peak resulting from enzyme activity (Fig. 4) was 48%. **b** endoxylanase on xylohexaose-containing arrays with DP6 + H 1036.9 m/z (100%), DP5 + H 904.9 m/z (1.2%), DP4 + H 772.9 m/z (0.6%), DP3 + H 640.8 m/z (2.1%) while peaks resulting from enzyme activity (Fig. 4) were 5.3%, 9.1% and 6.4% respectively.

Supplementary Table S1. qRT-PCR primers

Supplementary Table S2. Shotgun proteomics identification of proteins in the filtrate of *A. niger* cultures grown in the presence of wheat straw with Score: SEQUEST protein score, #PSMs: total number of identified peptide sequences.

Supplementary Table S3. a Chemical shifts of ^{1}H-^{1}H TOCSY NMR of fraction H8, **b** chemical shifts of ^{1}H and 2D ^{1}H-^{13}C HSQC NMR and **c** ratios of integrated ^{1}H signals.

Supplementary Table S4: Ratio of the intensities of MALDI-ToF MS signal, calculated for DP5/DP8, DP6/DP8, DP5/DP6 of fraction AX10 after labelling via reductive amination using various reaction times and procedures. *Procedure a*: Excess of oligosaccharide, hexadecylamine (limiting reagent), excess of $NaBH_3CN$, sodium borate/sodium sulfate buffer. *Procedure b*: Oligosaccharide (limiting reagent), excess of hexadecylamine, excess of $NaBH_3CN$, sodium borate/sodium sulfate buffer. *Procedure c*: Excess of oligosaccharide, hexadecylamine (limiting reagent), excess of $NaBH_3CN$, sodium borate buffer.

Supplementary Table S5: Interday reproducibility of array generation, given as mean with standard deviation of the ratio of the intensities of MALDI-ToF MS signal, calculated for DP5/DP8, DP6/DP8, DP5/DP6 of fraction AX10 and H9 immobilised over multiple days.

Fig. S1a

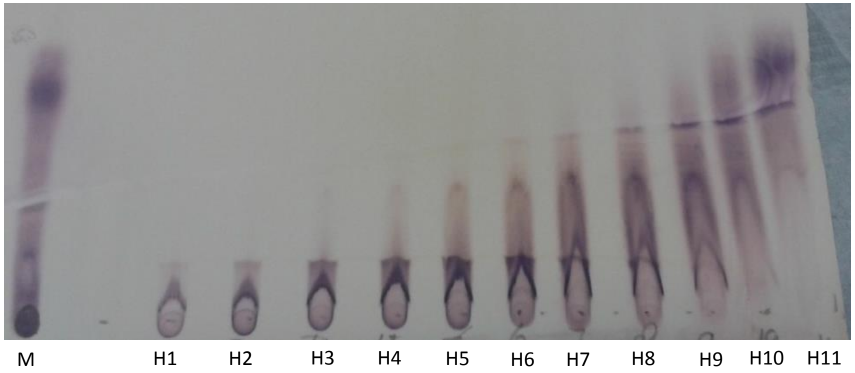
M
H1
H2
H3
H4
H5
H6
H7
H8
H9
H10
H11

b

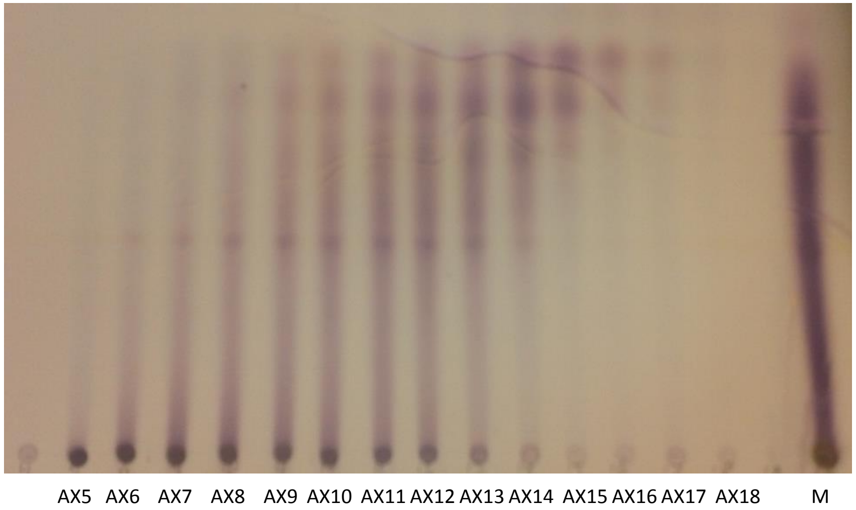
AX5
AX6
AX7
AX8
AX9
AX10
AX11
AX12
AX13
AX14
AX15
AX16
AX17
AX18
M

Fig. S1c

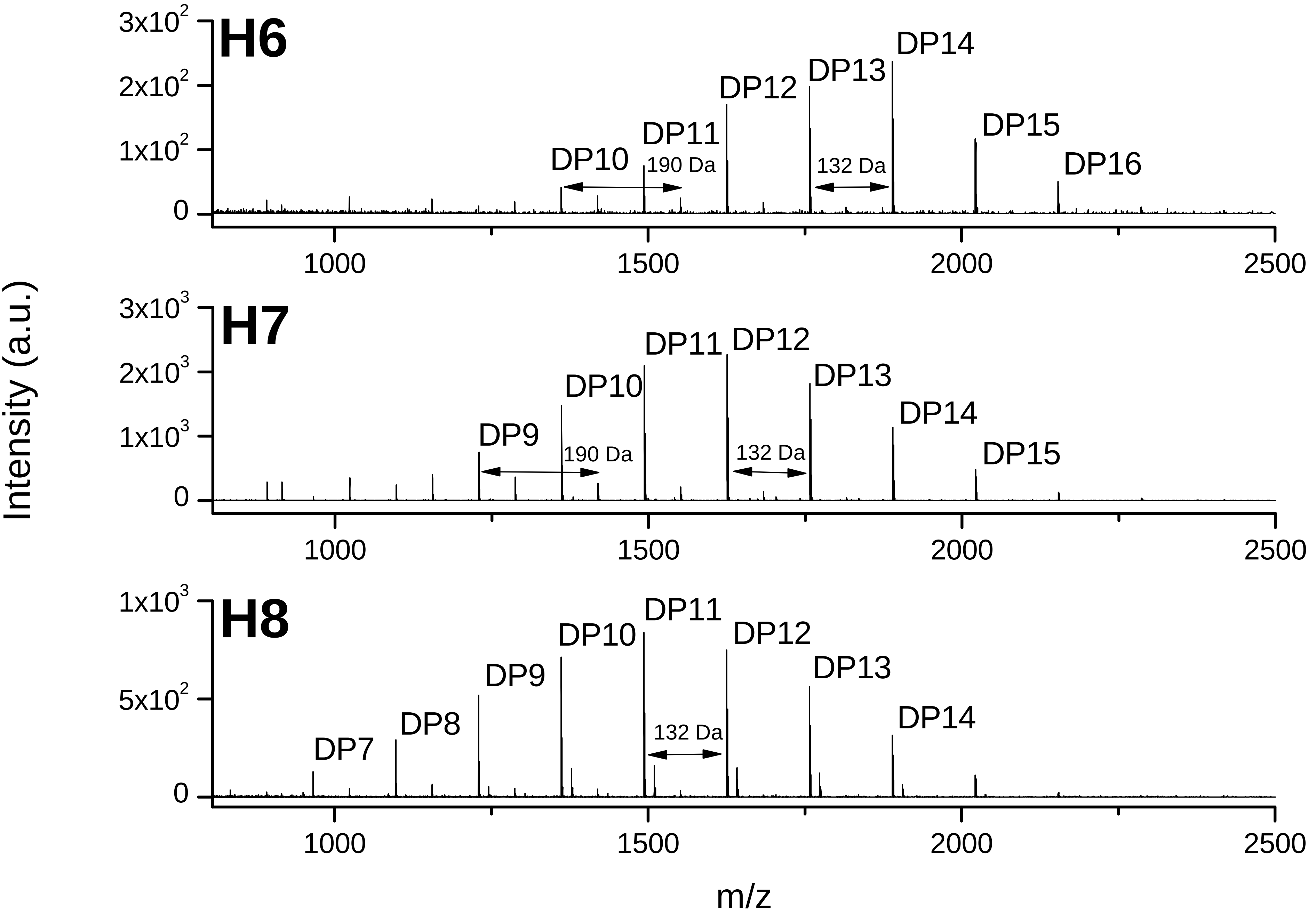
H6
DP10
DP11
DP12
DP13
DP14
DP15
DP16
190 Da
132 Da
H7
DP9
DP10
DP11
DP12
DP13
DP14
DP15
190 Da
132 Da
H8
DP7
DP8
DP9
DP10
DP11
DP12
DP13
DP14
132 Da
Intensity (a.u.)
m/z
3x10^2
2x10^2
1x10^2
0
3x10^3
2x10^3
1x10^3
1x10^3
5x10^2
1000
1500
2000
2500

Fig. S1d

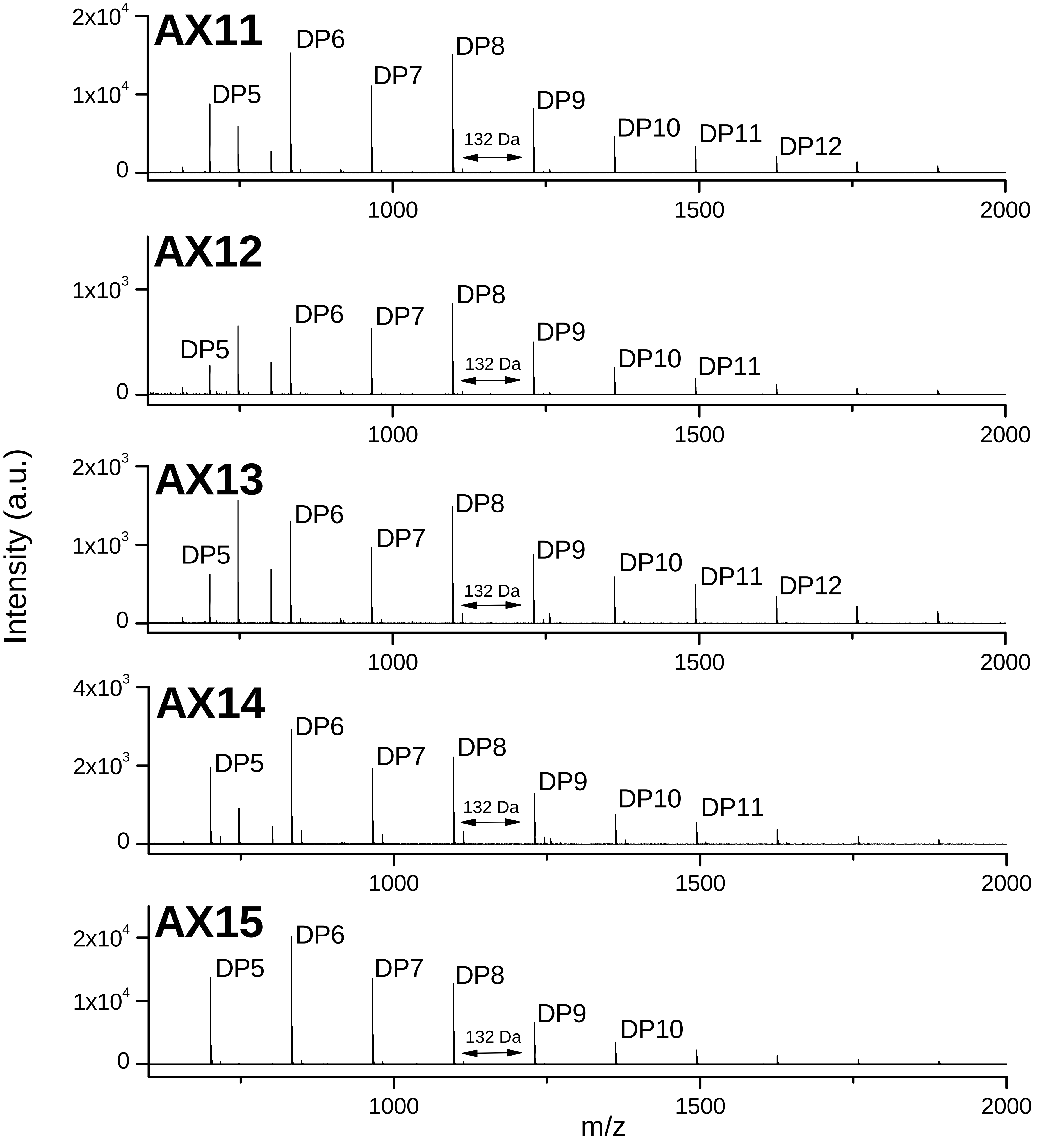
AX11
AX12
AX13
AX14
AX15
DP5
DP6
DP7
DP8
DP9
DP10
DP11
DP12
132 Da
2x10^4
1x10^4
1x10^3
2x10^3
4x10^3
0
1000
1500
2000
Intensity (a.u.)
m/z

Fig. S2a

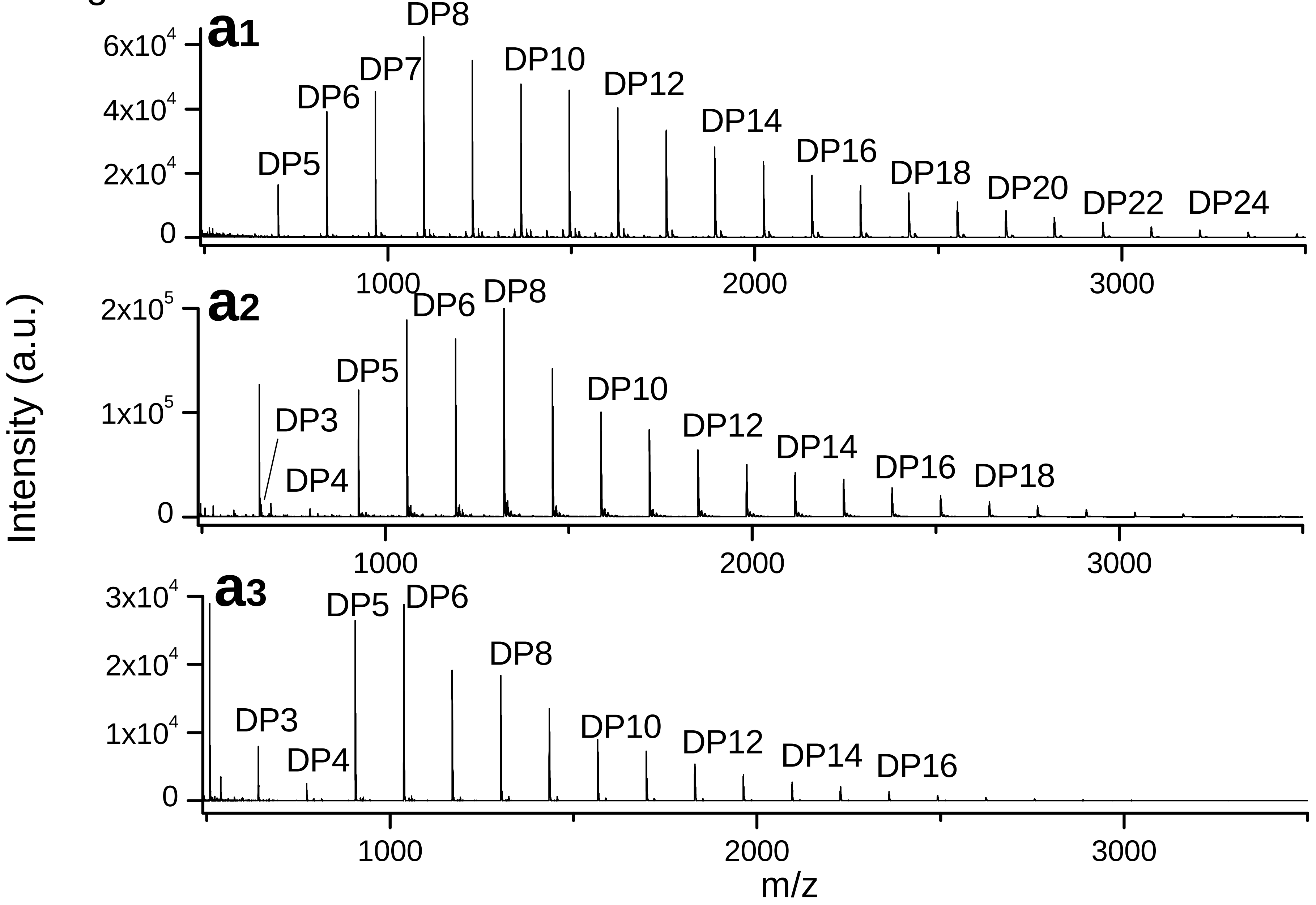

a1
DP5
DP6
DP7
DP8
DP10
DP12
DP14
DP16
DP18
DP20
DP22
DP24
6x10^4
4x10^4
2x10^4
0
1000
2000
3000
a2
DP3
DP4
DP5
DP6
DP8
DP10
DP12
DP14
DP16
DP18
2x10^5
1x10^5
0
1000
2000
3000
a3
DP3
DP4
DP5
DP6
DP8
DP10
DP12
DP14
DP16
3x10^4
2x10^4
1x10^4
0
1000
2000
3000
Intensity (a.u.)
m/z

Fig. S2b

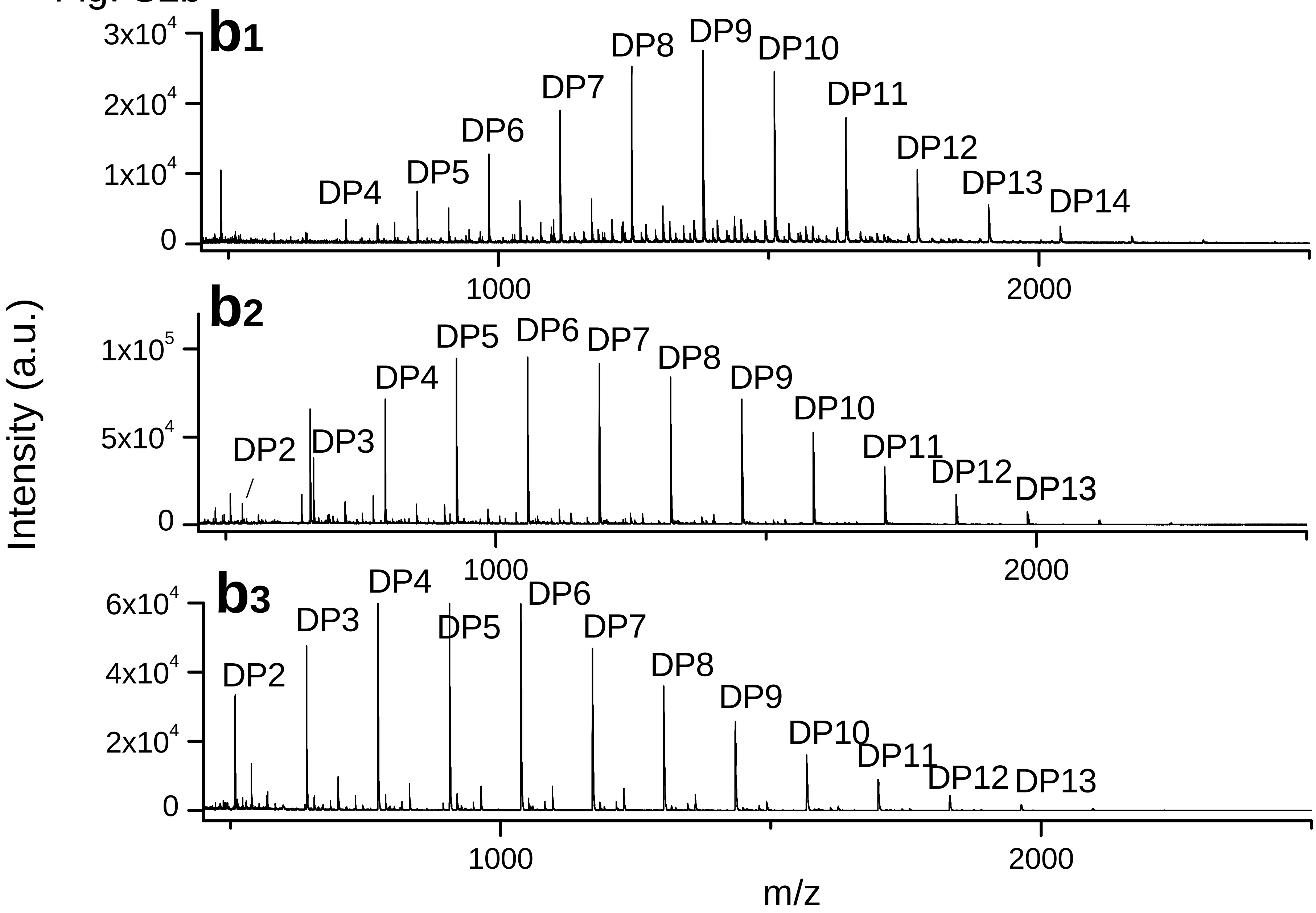

b1
3x10^4
2x10^4
1x10^4
0
DP4
DP5
DP6
DP7
DP8
DP9
DP10
DP11
DP12
DP13
DP14
1000
2000
b2
1x10^5
5x10^4
0
DP2
DP3
DP4
DP5
DP6
DP7
DP8
DP9
DP10
DP11
DP12
DP13
1000
2000
b3
6x10^4
4x10^4
2x10^4
0
DP2
DP3
DP4
DP5
DP6
DP7
DP8
DP9
DP10
DP11
DP12
DP13
1000
2000
Intensity (a.u.)
m/z

Fig. S3

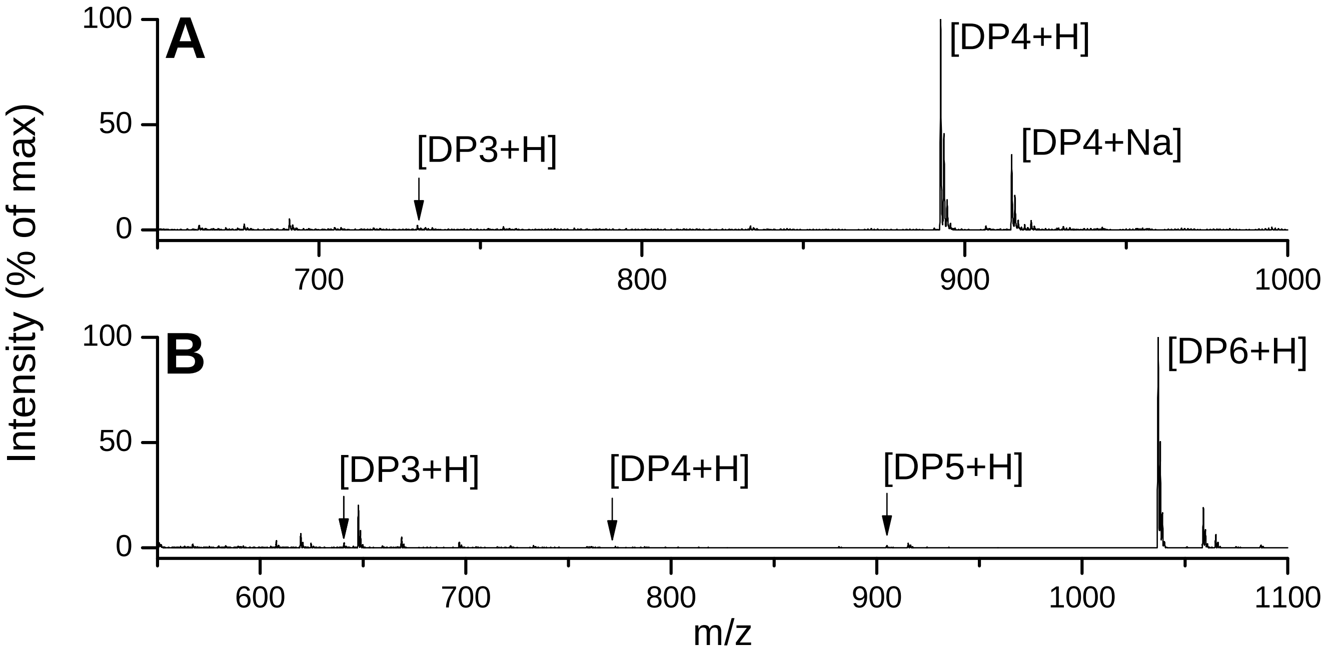

A
[DP4+H]
[DP4+Na]
[DP3+H]
B
[DP6+H]
[DP3+H]
[DP4+H]
[DP5+H]
Intensity (% of max)
100
50
0
700
800
900
1000
600
1100
m/z

Supplementary Table S1, primers used for qRT-PCR

gene name	gene ID	primer name	sequence (5'-3')	reference
actA	An15g00560	An15g00560Fw	TCCTGGGTCTGGAGAGCGGTG	van Munster, J. M. et al. *Fungal Genet Biol* (2014), 72:34-47
		An15g00560Rv	CTGCATACGGTCGGAGATACCGGG	van Munster, J. M. et al. *Fungal Genet Biol* (2014), 72:34-47
cbhA	An07g09330	cbhAFw	CCAGCAAGCCGGAACGCTCA	9
		cbhARv	AACGCGCCGTTTAGCCCACA	9
cbhB	An01g11660	cbhBFw	CCAGCGATGGCAGCTGCAC	9
		cbhBRv	CTGCCGGACGTGGTCACACC	9
xynB	An01g00780	xynBFw	CACGACTCTGTCGCCCAGCG	9
		xynBRv	GGGGGTCAGTGGTCCAGCCA	9

**Supplementary Table S2; Shotgun Proteomics of *A. niger* culture filtrates**					Biological replicate B				Biological replicate A			
Accession	Description	# AAs	MW [kDa]	calc. pI	Score	Coverage	# Unique Peptides	# PSMs	Score	Coverage	# Unique Peptides	# PSMs
A2QFV7	Probable endo-1,4-beta-xylanase C OS=Aspergillus niger (strain CBS 513.88 / FGSC A1513) GN=xlnC PE=1 SV=1 - [XYNC_ASPNC]	327	35.46	6.65	3836.167	87.77	38	1293	4386.343	87.77	38	1437
A2QPG2	Probable 1,4-beta-D-glucan cellobiohydrolase A OS=Aspergillus niger (strain CBS 513.88 / FGSC A1513) GN=cbhA PE=3 SV=1 - [CBHA_ASPNC]	452	48.22	4.32	2510.741	52.43	12	694	3181.424	52.43	12	863
A2Q7I0	Probable endo-1,4-beta-xylanase B OS=Aspergillus niger (strain CBS 513.88 / FGSC A1513) GN=xlnB PE=3 SV=1 - [XYNB_ASPNC]	225	24.04	5.45	2237.921	36.44	5	715	2421.212	36	4	759
A2QFV9	Probable alpha-L-arabinofuranosidase axhA OS=Aspergillus niger (strain CBS 513.88 / FGSC A1513) GN=axhA PE=3 SV=1 - [AXHA_ASPNC]	332	35.81	4.86	1629.407	61.45	13	502	2057.846	61.45	13	601
A2R511	Probable alpha-L-arabinofuranosidase B OS=Aspergillus niger (strain CBS 513.88 / FGSC A1513) GN=abfB PE=3 SV=1 - [ABFB_ASPNC]	499	52.48	4.44	1160.002	35.87	11	356	1273.708	35.87	11	376
A2QAI7	Probable 1,4-beta-D-glucan cellobiohydrolase B OS=Aspergillus niger (strain CBS 513.88 / FGSC A1513) GN=cbhB PE=3 SV=1 - [CBHB_ASPNC]	536	56.17	4.30	988.6004	34.51	10	328	1214.966	34.51	10	430
A2QSY5	Probable feruloyl esterase A OS=Aspergillus niger (strain CBS 513.88 / FGSC A1513) GN=faeA PE=3 SV=1 - [FAEA_ASPNC]	281	30.53	4.55	591.2076	44.48	10	207	735.6362	44.48	10	247
A2QA27	Probable exo-1,4-beta-xylosidase xlnD OS=Aspergillus niger (strain CBS 513.88 / FGSC A1513) GN=xlnD PE=3 SV=1 - [XYND_ASPNC]	804	87.16	4.89	410.8238	34.45	22	150	403.6227	34.58	22	149
A2QPC3	Probable endo-beta-1,4-glucanase B OS=Aspergillus niger (strain CBS 513.88 / FGSC A1513) GN=eglB PE=3 SV=1 - [EGLB_ASPNC]	331	36.54	4.55	332.6013	28.4	10	169	332.4204	31.42	10	178
A2RAL4	Probable beta-glucosidase A OS=Aspergillus niger (strain CBS 513.88 / FGSC A1513) GN=bglA PE=3 SV=1 - [BGLA_ASPNC]	860	93.17	4.78	307.6645	33.37	28	111	276.1129	36.16	29	101
A2QYU7	Probable feruloyl esterase C OS=Aspergillus niger (strain CBS 513.88 / FGSC A1513) GN=faeC PE=3 SV=1 - [FAEC_ASPNC]	270	28.57	5.02	233.4921	36.67	6	65	284.6185	36.67	6	72
A2QAS2	Probable mannosyl-oligosaccharide alpha-1,2-mannosidase 1B OS=Aspergillus niger (strain CBS 513.88 / FGSC A1513) GN=mns1B PE=3 SV=1 - [MNS1B	513	55.89	5.05	213.7005	31.97	14	64	225.6573	36.26	15	69
A2R2L1	Probable rhamnogalacturonate lyase A OS=Aspergillus niger (strain CBS 513.88 / FGSC A1513) GN=rglA PE=3 SV=2 - [RGLA_ASPNC]	531	57.10	4.60	208.3747	20.9	10	71	270.6945	22.22	9	90
A2R0E0	Extracellular exo-inulinase inuE OS=Aspergillus niger (strain CBS 513.88 / FGSC A1513) GN=inuE PE=2 SV=1 - [INUE_ASPNC]	537	59.11	5.43	197.594	38.73	16	88	141.211	34.45	13	60
A5ABF5	Probable beta-glucosidase M OS=Aspergillus niger (strain CBS 513.88 / FGSC A1513) GN=bglM PE=3 SV=1 - [BGLM_ASPNC]	765	82.07	4.74	163.5841	21.96	13	54	162.2629	23.66	14	57
A2R3L3	Aspartic protease pep1 OS=Aspergillus niger (strain CBS 513.88 / FGSC A1513) GN=pep1 PE=3 SV=1 - [PEPA_ASPNC]	394	41.25	4.67	131.3712	25.38	8	56	119.9708	24.62	8	48
A2R2S8	Cell wall protein phiA OS=Aspergillus niger (strain CBS 513.88 / FGSC A1513) GN=phiA PE=2 SV=1 - [PHIA_ASPNC]	184	19.17	5.03	120.3915	23.37	4	34	101.3156	25.54	5	32
A2QEJ9	Probable alpha-galactosidase B OS=Aspergillus niger (strain CBS 513.88 / FGSC A1513) GN=aglB PE=3 SV=1 - [AGALB_ASPNC]	443	48.80	5.08	107.8469	23.25	9	49	114.5235	19.64	6	53
A2QKT4	Probable mannan endo-1,4-beta-mannosidase A OS=Aspergillus niger (strain CBS 513.88 / FGSC A1513) GN=manA PE=1 SV=1 - [MANA_ASPNC]	383	41.22	4.61	104.2714	31.59	7	32	118.8116	30.29	6	32
A2QAN3	Probable beta-galactosidase A OS=Aspergillus niger (strain CBS 513.88 / FGSC A1513) GN=lacA PE=3 SV=1 - [BGALA_ASPNC]	1007	109.64	5.21	77.29582	20.56	15	33	71.64827	21.75	15	32
A2QH21	Probable glucan endo-1,3-beta-glucosidase eglC OS=Aspergillus niger (strain CBS 513.88 / FGSC A1513) GN=eglC PE=3 SV=2 - [EGLC_ASPNC]	460	46.32	4.49	74.7548	19.57	5	25	64.61966	19.57	5	24
A2QK83	Probable endo-xylogalacturonan hydrolase A OS=Aspergillus niger (strain CBS 513.88 / FGSC A1513) GN=xghA PE=3 SV=1 - [XGHA_ASPNC]	406	42.10	4.51	72.05174	17.24	6	30	58.36289	17.24	6	23
A2RB93	Probable arabinogalactan endo-beta-1,4-galactanase A OS=Aspergillus niger (strain CBS 513.88 / FGSC A1513) GN=galA PE=3 SV=1 - [GANA_ASPNC]	350	38.67	4.07	64.57549	24.29	7	20	88.52927	24.29	6	25
A2R2S6	Probable alpha-galactosidase D OS=Aspergillus niger (strain CBS 513.88 / FGSC A1513) GN=aglD PE=3 SV=2 - [AGALD_ASPNC]	660	71.18	4.83	61.81282	16.97	10	24	50.46856	19.7	11	25
A2RAR6	Probable glucan 1,3-beta-glucosidase A OS=Aspergillus niger (strain CBS 513.88 / FGSC A1513) GN=exgA PE=3 SV=1 - [EXGA_ASPNC]	416	45.50	5.19	58.95625	25.24	9	23	49.66892	27.88	8	19
A2R3X3	Probable alpha-glucuronidase A OS=Aspergillus niger (strain CBS 513.88 / FGSC A1513) GN=aguA PE=3 SV=1 - [AGUA_ASPNC]	841	93.67	5.38	55.70176	20.57	15	36	80.58374	22.47	16	38
A2QEQ6	Beta-glucuronidase OS=Aspergillus niger (strain CBS 513.88 / FGSC A1513) GN=An02g11890 PE=1 SV=1 - [GUS79_ASPNC]	541	58.82	5.47	49.53932	20.52	8	17	50.59749	16.82	7	15
A2R0Z6	Probable feruloyl esterase B OS=Aspergillus niger (strain CBS 513.88 / FGSC A1513) GN=faeB PE=3 SV=1 - [FAEB_ASPNC]	521	57.17	5.20	48.00242	14.01	5	14	54.39299	15.55	6	16
A2QYR9	Probable 1,4-beta-D-glucan cellobiohydrolase C OS=Aspergillus niger (strain CBS 513.88 / FGSC A1513) GN=cbhC PE=3 SV=1 - [CBHC_ASPNC]	459	48.10	4.53	45.85379	10.89	3	27	45.22075	18.52	4	23
A2Q7E0	Probable alpha-L-arabinofuranosidase A OS=Aspergillus niger (strain CBS 513.88 / FGSC A1513) GN=abfA PE=3 SV=1 - [ABFA_ASPNC]	628	67.91	4.30	45.31327	13.22	6	15	29.4525	13.22	6	12
A2QT85	Probable arabinan endo-1,5-alpha-L-arabinosidase A OS=Aspergillus niger (strain CBS 513.88 / FGSC A1513) GN=abnA PE=3 SV=1 - [ABNA_ASPNC]	321	34.46	4.22	42.86552	4.67	2	14	37.9504	4.67	2	11
A2QK82	Probable pectinesterase A OS=Aspergillus niger (strain CBS 513.88 / FGSC A1513) GN=pmeA PE=3 SV=1 - [PMEA_ASPNC]	327	34.60	4.39	42.15743	19.57	4	18	59.39917	23.24	5	24
A2R060	Probable exopolygalacturonase X OS=Aspergillus niger (strain CBS 513.88 / FGSC A1513) GN=pgaX PE=3 SV=1 - [PGLRX_ASPNC]	435	47.26	4.82	39.48248	15.63	5	13	22.17836	13.56	3	10
A2QHG0	Probable exopolygalacturonase B OS=Aspergillus niger (strain CBS 513.88 / FGSC A1513) GN=pgxB PE=3 SV=1 - [PGXB_ASPNC]	438	48.35	4.86	39.31279	22.6	9	18	25.81601	18.95	7	14
A2R797	Probable alpha-fucosidase A OS=Aspergillus niger (strain CBS 513.88 / FGSC A1513) GN=afcA PE=3 SV=1 - [AFCA_ASPNC]	793	86.51	4.91	24.53108	16.52	12	15	22.9456	11.6	7	10
A5AAG2	Probable arabinan endo-1,5-alpha-L-arabinosidase C OS=Aspergillus niger (strain CBS 513.88 / FGSC A1513) GN=abnC PE=3 SV=1 - [ABNC_ASPNC]	318	34.03	4.87	24.49346	10.69	4	12	NA	NA	NA	NA
A2RAY7	Putative galacturan 1,4-alpha-galacturonidase C OS=Aspergillus niger (strain CBS 513.88 / FGSC A1513) GN=rgxC PE=2 SV=1 - [RGXC_ASPNC]	423	45.87	5.20	22.18053	16.55	4	9	20.71233	11.58	3	6
A2Q877	Probable xyloglucan-specific endo-beta-1,4-glucanase A OS=Aspergillus niger (strain CBS 513.88 / FGSC A1513) GN=xgeA PE=3 SV=1 - [XGEA_ASPNC]	241	25.47	3.88	21.76691	14.11	2	11	21.61399	14.11	2	12
A2QA64	Probable beta-galactosidase B OS=Aspergillus niger (strain CBS 513.88 / FGSC A1513) GN=lacB PE=3 SV=2 - [BGALB_ASPNC]	1017	111.74	5.00	21.22608	6	6	9	15.32841	5.9	6	8
A2QV36	Probable pectate lyase A OS=Aspergillus niger (strain CBS 513.88 / FGSC A1513) GN=plyA PE=3 SV=1 - [PLYA_ASPNC]	323	34.30	4.82	19.75821	13.62	4	14	28.6039	13.62	4	15
A2QAH3	Probable endopolygalacturonase I OS=Aspergillus niger (strain CBS 513.88 / FGSC A1513) GN=pgaI PE=3 SV=1 - [PGLR1_ASPNC]	368	38.09	4.48	19.3904	13.32	4	11	22.07549	11.96	3	9
A2QW66	Probable exopolygalacturonase A OS=Aspergillus niger (strain CBS 513.88 / FGSC A1513) GN=pgxA PE=3 SV=1 - [PGXA_ASPNC]	434	46.94	4.79	18.59541	10.14	5	10	22.42316	10.14	5	10
A2QYE5	Probable rhamnogalacturonase A OS=Aspergillus niger (strain CBS 513.88 / FGSC A1513) GN=rhgA PE=3 SV=1 - [RHGA_ASPNC]	446	46.93	4.23	16.74227	15.02	4	7	24.95915	5.83	2	8
A2QL72	Probable alpha-galactosidase A OS=Aspergillus niger (strain CBS 513.88 / FGSC A1513) GN=aglA PE=3 SV=1 - [AGALA_ASPNC]	537	59.18	5.03	16.61905	11.92	5	10	18.05999	5.4	3	8
A2R3I1	Probable pectin lyase A OS=Aspergillus niger (strain CBS 513.88 / FGSC A1513) GN=pelA PE=3 SV=1 - [PELA_ASPNC]	379	39.76	4.34	16.34141	16.09	5	11	14.46537	8.97	3	9
A2QTU5	Alpha-xylosidase A OS=Aspergillus niger (strain CBS 513.88 / FGSC A1513) GN=axlA PE=1 SV=1 - [XYLA_ASPNC]	736	82.54	5.66	14.92676	13.04	7	10	22.78718	13.18	7	12
A2QEW2	Probable exopolygalacturonase C OS=Aspergillus niger (strain CBS 513.88 / FGSC A1513) GN=pgxC PE=3 SV=2 - [PGXC_ASPNC]	440	47.82	5.20	14.81849	9.09	3	6	NA	NA	NA	NA
A2QWU9	Beta-mannosidase A OS=Aspergillus niger (strain CBS 513.88 / FGSC A1513) GN=mndA PE=3 SV=1 - [MANBA_ASPNC]	931	103.96	5.29	14.74085	12.14	7	11	6.310669	5.91	4	4
P0C953	Histone H2A OS=Aspergillus niger (strain CBS 513.88 / FGSC A1513) GN=httA PE=3 SV=1 - [H2A_ASPNC]	134	14.14	10.55	8.224466	11.94	2	4	8.499234	11.94	2	4
A2R2W3	Probable cutinase 1 OS=Aspergillus niger (strain CBS 513.88 / FGSC A1513) GN=An14g02170 PE=3 SV=1 - [CUTI1_ASPNC]	262	26.67	4.88	7.27599	9.92	2	3	10.04579	9.92	2	3
A2QCV8	Probable endopolygalacturonase B OS=Aspergillus niger (strain CBS 513.88 / FGSC A1513) GN=pgaB PE=3 SV=1 - [PGLRB_ASPNC]	362	37.80	6.77	7.110866	9.67	3	4	4.506392	6.63	2	2
A2QUQ2	Endochitinase A OS=Aspergillus niger (strain CBS 513.88 / FGSC A1513) GN=ctcA PE=2 SV=1 - [CHIA1_ASPNC]	1257	122.02	4.55	5.929324	0.88	2	3	4.313844	0.88	2	2
A2QY49	Histone H2B OS=Aspergillus niger (strain CBS 513.88 / FGSC A1513) GN=htb1 PE=3 SV=1 - [H2B_ASPNC]	141	14.99	10.13	4.44716	18.44	3	4	4.437262	13.48	2	3
A2QE32	40S ribosomal protein S0 OS=Aspergillus niger (strain CBS 513.88 / FGSC A1513) GN=rps0 PE=3 SV=1 - [RSSA_ASPNC]	298	31.93	4.91	4.07477	6.71	2	2	NA	NA	NA	NA
A5ABH4	Probable rhamnogalacturonate lyase B OS=Aspergillus niger (strain CBS 513.88 / FGSC A1513) GN=rglB PE=3 SV=1 - [RGLB_ASPNC]	706	78.37	5.11	2.444819	4.25	3	4	0	2.97	2	4

	Chemical shift (p.p.m.)	
**Protons**	**Internal β-Xyl*p***	**Non-reducing terminal β-Xyl*p***
^{1}H	4.48	4.46
^{2}H	3.29	3.26
^{3}H	3.556	3.42
^{4}H	3.79	3.62
^{5}H	4.10, 3.38	3.97, 3.31

Table S3a, Upfield ^{1}H chemical shifts of xylose oligosaccharide as determined from the 2D TOCSY spectrum of fraction H8

	Chemical shift (p.p.m.)			
	Fraction AX14		Fraction H8	
**Residue**	**^{1}H (1D)**	**^{13}C (HSQC)**	**^{1}H (1D)**	**^{13}C (HSQC)**
α-Xyl*p*^{1}	5.184	93.1	5.183	92.9
β-Xyl*p*^{1}	4.584	97.6	4.584	97.6
β-Xyl*p*$^{2/3/4/5}$	~4.47	~102.7	~4.48	~102.8
α-Ara*f*-X^{N}-*O*3s	5.395	108.7	NA	NA
α-Ara*f*-X^{N}-*O*-2	5.224	109.8	NA	NA
α-Ara*f*-X^{N}-*O*-3d	5.273	109.2	NA	NA
β-Xyl*p*^{N}-A*f*-*O*-2,3	4.640	100.9	NA	NA
β-Xyl*p*^{N}-A*f*-*O*-3	4.43-4.45	NR	NA	NA
4-*O*-Me-α-GlcA-X^{N}-2	NA	NA	5.285	~98.7
α-GlcA-X^{N}-2	NA	NA	5.303	~98.7
β-Xyl*p*^{N}-GlcA	NA	NA	4.641	~102.7
β-Xyl*p*^{N}-MeGlcA	NA	NA	4.623	~102.7

Table S3b, Assignment of chemical shifts of ^{1}H and ^{1}H-^{13}C HSQC NMR anomeric resonances. Xyl*p*^{1} is reducing end xylopyranose; β-Xyl*p*$^{2/3/4/5}$ is the total of unsubstituted Xyl*p* residues other than the reducing end; α-Ara*f*-X^{N}-*O*- single (3s) or double (2 and 3d) arabinofuranosidase decoration on unidentified Xyl*p* via α-1,3-, or α-1,2- and α-1,3- linkage respectively; β-Xyl*p*^{N}-A*f*-*O*-3/2,3 Xyl*p* with single/double Ara*f* decoration respectively; (4-*O*-Me)-α-GlcA-X^{N}-C2 signal for GlcA and 4MeGlcA attached via α-1,2 to an unidentified Xyl*p*, β-Xyl*p*^{N}-(Me)GlcA unidentified Xyl*p* decorated with GlcA or 4MeGlcA, NA not identified in this fraction, NR not resolvable.

	Fraction				
**Signal integration ratio ^{1}H (1D)**	**H6**	**H7**	**H8**	**AX14**	**AX15**
Xyl*p*^{1}	1	1	1	1	1
β-Xyl*p*$^{2/3/4/5}$	8.2	6.5	6.3	NR	NR
(4-*O*-Me)-α-GlcA-X^{N}-C2	1.1	0.7	0.6	NA	NA
β-Xyl*p*^{N}-(Me)GlcA	1.2	0.8	0.6	NA	NA
β-Xyl*p*^{N}-A*f*-*O*-2,3	NA	NA	NA	0.4	0.3
β-Xyl*p*^{N}-A*f*-*O*-3 + β-Xyl*p*$^{2/3/4/5}$	NA	NA	NA	3.5	3.0
α-Ara*f*-X^{N}-*O*-3s	NA	NA	NA	0.9	0.7
α-Ara*f*-X^{N}-*O*-2	NA	NA	NA	0.4	0.3
α-Ara*f*-X^{N}-*O*-3d	NA	NA	NA	0.4	0.3

Table S3c, Signal integration ratios ^{1}H NMR. With the sum of the signal for the α- and β-anomer of the reducing end xylopyranose (Xyl*p*^{1}) set to 1, β-Xyl*p*^{N}-A*f*-*O*-3 + β-Xyl*p*$^{2/3/4/5}$combined ratio for β-Xyl*p*^{N}-A*f*-*O*-3 and β-Xyl*p*$^{2/3/4/5}$, other notations as in Table S3b.

Supplementary Table S4

	DP5/DP8			DP6/DP8			DP5/DP6		
Procedure	***a***	***b***	***c***	***a***	***b***	***c***	***a***	***b***	***c***
1 hour	0.32	0.40	0.35	0.70	0.79	0.74	0.46	0.52	0.47
2 hours	0.39	0.39	0.38	0.77	0.80	0.76	0.50	0.50	0.50
4 hours	0.54 ± 0.02	0.37	0.40	0.92 ± 0.02	0.81	0.85	0.59 ± 0.01	0.48	0.47
8 hours	0.55	0.36	0.47	0.92	0.77	0.89	0.60	0.47	0.53
1 day	0.56	0.35	0.52	0.93	0.75	0.99	0.60	0.46	0.52
3 days	0.56	0.33	0.55	0.92	0.74	0.95	0.60	0.45	0.53
Unlabelled oligosaccharide	0.24			0.53			0.45		

Supplementary Table S5

	DP5/DP8	DP6/DP8	DP5/DP6
AX10 day 1	1.71	1.82	0.94
AX10 day 2	1.77	1.81	0.99
AX10 day 3	1.75	1.81	0.99
AX10 variability	1.74 ± 0.03	1.81 ± 0.01	0.97 ± 0.03
H9 day 1	1.93	1.68	1.15
H9 day 2	1.93	1.62	1.19
H9 day 3	1.97	1.65	1.24
H9 variability	1.94 ± 0.02	1.65 ± 0.03	1.19 ± 0.05
